# hMOF induces cisplatin resistance of ovarian cancer by regulating the stability and expression of MDM2

**DOI:** 10.1038/s41420-023-01478-y

**Published:** 2023-06-08

**Authors:** Mingbo Cai, Sulong Xu, Yuxi Jin, Jingjing Yu, Shan Dai, Xiao-Jing Shi, Ruixia Guo

**Affiliations:** 1 Department of Gynecology, The First Affiliated Hospital of Zhengzhou University, Zhengzhou, 450052 Henan China; 2 Laboratory Animal Center, Academy of Medical Sciences, Zhengzhou University, Zhengzhou, 450052 Henan China

**Keywords:** Ovarian cancer, Prognostic markers, Acetylation

## Abstract

Histone acetyltransferase human males absent on the first (hMOF) is a member of MYST family which participates in posttranslational chromatin modification by controlling the acetylation level of histone H4K16. Abnormal activity of hMOF occurs in multiple cancers and biological alteration of hMOF expression can affect diverse cellular functions including cell proliferation, cell cycle progression and embryonic stem cells (ESCs) self-renewal. The relationship between hMOF and cisplatin resistance was investigated in The Cancer Genome Atlas (TCGA) and Genomics of Drug Sensitivity in Cancer (GDSC) database. Lentiviral-mediated hMOF-overexpressed cells or hMOF-knockdown cells were established to investigate its role on cisplatin-based chemotherapy resistance in vitro ovarian cancer cells and animal models. Furthermore, a whole transcriptome analysis with RNA sequencing was used to explore the underlying molecular mechanism of hMOF affecting cisplatin-resistance in ovarian cancer. The data from TCGA analysis and IHC identification demonstrated that hMOF expression was closely associated with cisplatin-resistance in ovarian cancer. The expression of hMOF and cell stemness characteristics increased significantly in cisplatin-resistant OVCAR3/DDP cells. In the low hMOF expressing ovarian cancer OVCAR3 cells, overexpression of hMOF improved the stemness characteristics, inhibited cisplatin-induced apoptosis and mitochondrial membrane potential impairment, as well as reduced the sensitivity of OVCAR3 cells to cisplatin treatment. Moreover, overexpression of hMOF diminished tumor sensitivity to cisplatin in a mouse xenograft tumor model, accompanied by decrease in the proportion of cisplatin-induced apoptosis and alteration of mitochondrial apoptosis proteins. In addition, opposite phenotype and protein alterations were observed when knockdown of hMOF in the high hMOF expressing ovarian cancer A2780 cells. Transcriptomic profiling analysis and biological experimental verification orientated that MDM2-p53 apoptosis pathway was related to hMOF-modulated cisplatin resistance of OVCAR3 cells. Furthermore, hMOF reduced cisplatin-induced p53 accumulation by stabilizing MDM2 expression. Mechanistically, the increased stability of MDM2 was due to the inhibition of ubiquitinated degradation, which resulted by increased of MDM2 acetylation levels by its direct interaction with hMOF. Finally, genetic inhibition MDM2 could reverse hMOF-mediated cisplatin resistance in OVCAR3 cells with up-regulated hMOF expression. Meanwhile, treatment with adenovirus expressing shRNA of hMOF improved OVCAR3/DDP cell xenograft sensitivity to cisplatin in mouse. Collectively, the results of the study confirm that MDM2 as a novel non-histone substrate of hMOF, participates in promoting hMOF-modulated cisplatin chemoresistance in ovarian cancer cells. hMOF/MDM2 axis might be a potential target for the treatment of chemotherapy-resistant ovarian cancer.

## Introduction

Ovarian cancer (OC) is one of the most common types of malignancies in women with high rates of morbidity and mortality [1, 2]. The main clinically used detection strategies for OC, including detailed gynecological evaluation, transvaginal ultrasound, and biomarker assays, have shown low predictive values with early-stage patients, as a result that most patients are diagnosed at advanced stages followed by poor outcomes [3]. Surgery and platinum-based chemotherapy are the standard treatments for newly diagnosed OC, but after a period of pharmacological interventions, platinum resistance inevitably develops [4, 5], which contributes a major obstacle to curative of this cancer. Hence, in-depth understanding of the mechanism of platinum resistance and searching for new therapeutic ways to overcome platinum resistance are urgent and creative areas of exploration in OC.

Histone acetyltransferases (HATs) and deacetylases (HDACs) are two opposing enzymes which contribute to regulation of the levels of acetylated histones [6]. Human males absent on the first (hMOF), is a component of MYST (MOZ, Ybf2, Sas2, and Tip60) family of HATs, specifically transfers an acetyl group from acetyl coenzyme A to histone H4 at lysine 16 (H4K16) [7, 8]. The increased acetylation of H4K16 could inhibit the formation of compact higher-order chromatin, leading to decompact nucleosomes and enhanced transcriptional activity [9]. Increasing research evidence indicates that hMOF not only participates in the regulation of gene transcription [10], chromatin composition, cell proliferation and differentiation [11], DNA damage repair [12] and other physiological processes, but also in the regulation of malignant tumor cell proliferation, apoptosis, and embryonic stem cells stemness [13]. Previous studies revealed hMOF was overexpressed in non-small-cell lung cancer (NSCLC) as an oncogene associated with large tumor size, advanced disease stage, metastasis and led to poor prognosis with drug and radiotherapy resistance [14, 15]. In a study of hepatocellular carcinoma, hMOF was shown to promote vascular invasion of tumor cells [16]. However, hMOF downregulation was considered an independent risk factor for patient prognosis in primary breast carcinoma and medulloblastoma [17]. Other studies had shown that the significant reduction of hMOF gene expression was observed in colorectal carcinoma, gastric cancer, and renal cell carcinoma [18], meanwhile, patients with high hMOF expression had higher overall survival rates in gastric cancer [19]. These studies mainly focused on the role of hMOF in tumor growth, metastasis and prognosis, however, little was known about its role in chemotherapy sensitivity and related mechanisms. A study in NSCLC indicated that interfering the expression of hMOF increased the sensitivity to cisplatin, 5-FU and bleomycin [14]. Additionally, knocking down hMOF in arsenic trioxide (As_2_O_3_)-exposed Hela cells caused cell damage and death [20]. On the contrary, overexpression of hMOF could promote VP16-induced cell death in H157 cells [21].

As we all know, the p53 tumor suppressor is an indispensable protein for inducing cell cycle arrest and apoptosis in response to DNA damage and cellular stress [22, 23]. Mouse double minute 2 homolog (MDM2), as an E3 ubiquitin ligase, is best characterized for binding and ubiquitinating the p53 protein, targeting p53 for proteasomal degradation [24]. MDM2 is overexpressed in most cancers, and associated with chemotherapy resistance [25]. CREB-binding protein (CBP) promotes the MDM2 acetylation, and an MDM2 mutant, mimicking the acetylated state, shows impaired ability to promote p53 degradation through ubiquitinating p53 protein [26]. Acetyltransferase p300 acetylates MDM2 protein, and then acetylation of MDM2 blocks its self-ubiquitination and triggers p53 ubiquitination. In response to DNA damage, MDM2 acetylation destabilizes p53 and subsequently inhibits cellular apoptosis [27].

Our previous study found that the expression of hMOF protein in OC tissues was significantly lower than that in benign ovarian tumors and normal ovarian tissues [28]. hMOF may enhance or decrease drug-sensitivity through the different mechanisms. Whether hMOF affects cisplatin sensitivity of OC cells and the related mechanisms are still unclear. Here, this study mainly focused on exploring the correlation between hMOF expression and cisplatin-resistance in OC cells. After interfering or promoting hMOF expression in OC cells, cisplatin sensitivity of OC cells and relevant regulatory mechanisms were investigated. More deeply, we revealed that hMOF could improve the acetylation and stability of MDM2 protein, thereby accelerating the degradation of p53 and promoting anti-apoptotic activity of OC cells. Finally, interference with hMOF expression could enhance the sensitivity of platinum-resistant OC cells to cisplatin in vivo tumor model.

## Results

### Elevated hMOF is closely associated with cisplatin-resistance in ovarian cancer

**Fig. 1:**
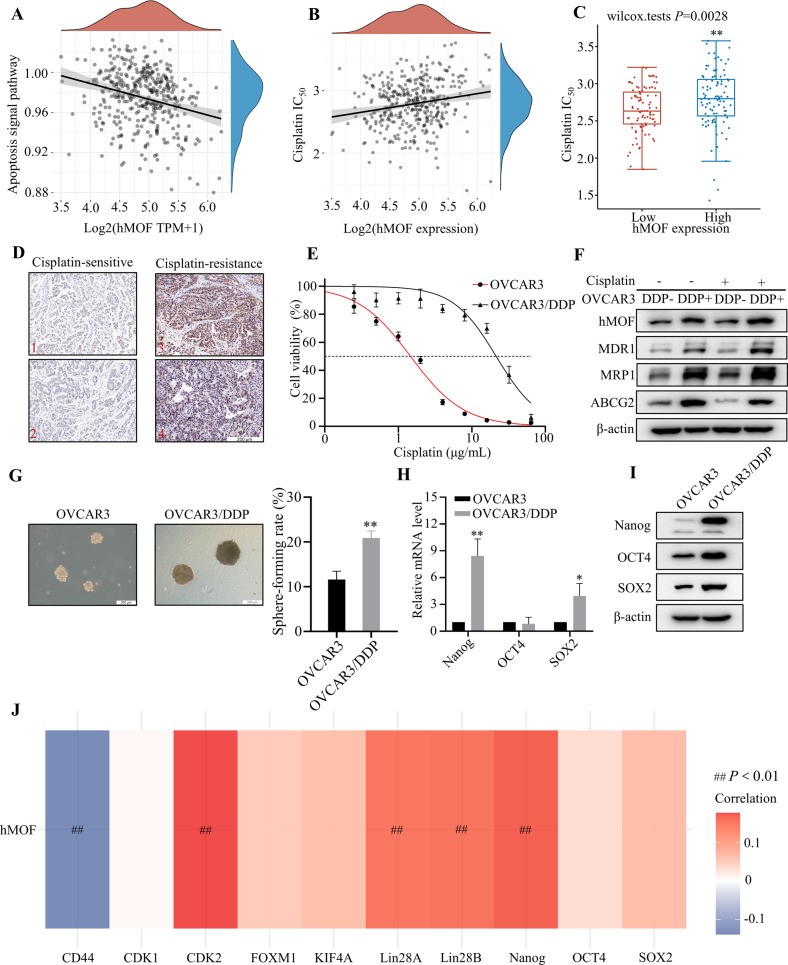
hMOF correlates with cisplatin-resistance in ovarian cancer. **A** Correlation analysis of hMOF expression with apoptosis signal pathway from TCGA database. **B** Relationship between hMOF expression and cisplatin response predicted by GDSC from 376 OC patients. **C** Predicted response analysis of cisplatin in 94 OC patients with low-expressed hMOF and 94 OC patients with high-expressed hMOF. **D** Representative images of hMOF immunostaining in cisplatin-sensitive and cisplatin-resistant ovarian cancer tissue samples. **E** The inhibitory effect of cisplatin to OVCAR3/DDP and OVCAR3 cells tested by MTT assay. **F** Western blot analysis of hMOF and drug resistance related proteins MDR1, MRP1, and ABCG2 expressions in OVCAR3 and OVCAR3/DDP cells treated with cisplatin. **G** Representative images of formed tumor spheres and quantification of sphere-forming rate. The mRNA (**H**) and protein (**I**) levels of Nanog, OCT4, and SOX2 in OVCAR3 and OVCAR3/DDP cells. **J** The correlations between hMOF and ESC markers from TCGA. Red represents positive correlation and blue represents negative correlation. *P < 0.05 and **P < 0.01 showed significantly difference in the compared two groups. ^##^*P* < 0.01 suggested that the two genes were significantly correlated.

To explore the effect of hMOF on cisplatin-resistance in OC, the correlation between hMOF and apoptosis signal pathway was analyzed from TCGA database. It was revealed that hMOF may inhibit cell apoptosis signaling pathway (Spearman = −0.24, *P* < 0.01) (Fig. 1A). The relationship between hMOF and cisplatin response in OC was further verified by GDSC database, it was observed that hMOF demonstrated a positive correlation with cisplatin IC_50_ (Spearman = 0.18, *P* < 0.01) (Fig. 1B) and high expression data of hMOF showed lower cisplatin sensitivity compared to low expression data (*P* < 0.01) (Fig. 1C). It was suggested that hMOF induced cisplatin resistance in OC may be responsible for the decrease in apoptosis. In clinical ovarian tumor samples, the expressions of hMOF in cisplatin-resistant tumors were higher than that in cisplatin-sensitive tumors (Fig. 1D). To further study the specific mechanisms of cisplatin-resistance in OC, cisplatin-resistant OVCAR3 cell line was constructed and named OVCAR3/DDP cells. As shown in Fig. 1E, the concentrations of cisplatin giving the half cell viability rates (IC_50_) were respectively 21.41 μg/mL for OVCAR3/DDP cells, and 1.46 μg/mL for parental OVCAR3 cells. The resistance index (RI) of OVCAR3/DDP cells was 14.63. The protein expression of hMOF in cisplatin-resistant OVCAR3/DDP cells was obviously elevated compared to OCVAR3 cells. The members of ATP-binding cassette transporter family MDR1, MRP1 and ABCG2, admittedly known as drug resistance related proteins [29], showed significant upregulation in OVCAR3/DDP cells (Fig. 1F). Increasing academic views considered that stemness of tumor cells had been one of the most important mechanisms leading to the chemotherapy resistance in tumors [30, 31]. To further evaluate whether cisplatin-resistance was associated with the stemness characteristics in OC, a sphere formation assay was performed. The volume of the formed spheres and sphere-forming rate were both increased in OVCAR3/DDP cells compared with OVCAR3 cells (Fig. 1G). Meanwhile, the mRNA levels of the core transcription factors of embryonic stem cells (ESC) Nanog and SOX2 were markedly higher in OVCAR3/DDP, however, the OCT4 mRNA level didn’t show significantly different between the OVCAR3 and OVCAR3/DDP cells (Fig. 1H). Then, western blot results indicated that the expressions of Nanog, OCT4, and SOX2 proteins were up-regulated in OVCAR3/DDP cells (Fig. 1I). The TCGA database analysis further validated a positive correlation between hMOF and stemness-related genes including Nanog, OCT4 and SOX2 in OC (Fig. 1J). These above experimental data suggested that high-expressed hMOF was related to cisplatin-resistance and stemness characteristics in OC.

### hMOF improves the stemness characteristics and cisplatin-resistance in ovarian cancer cells

**Fig. 2:**
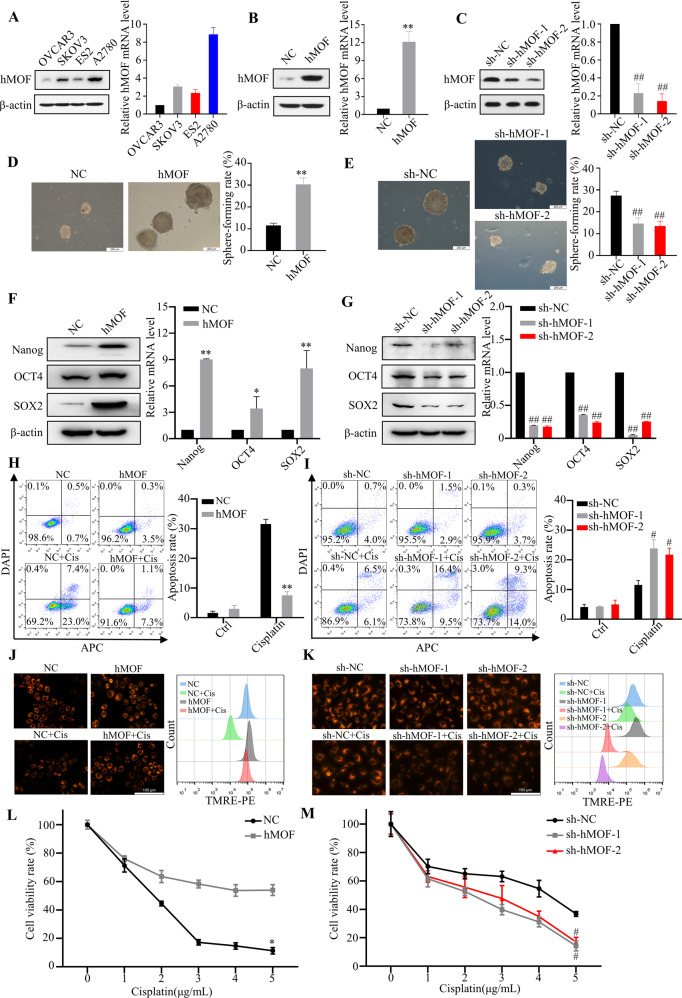
hMOF improves the stemness characteristics and cisplatin-resistance in vitro OC cells. **A** The protein and mRNA levels of hMOF in ovarian cancer cell lines OVCAR3, SKOV3, ES2, and A2780. **B** OVCAR3 cells were transfected with lentivirus containing hMOF gene and the expressions of hMOF were verified by western blot and qRT-PCR. **C** A2780 cells were transfected with lentivirus shMOF gene and the expressions of hMOF were verified by western blot and qRT-PCR. Representative images of formed tumor spheres and quantification of sphere-forming rate in NC, hMOF OVCAR3 cells (**D**) and sh-NC, sh-hMOF-1, sh-hMOF-2 A2780 cells (**E**). The protein and mRNA levels of Nanog, OCT4, and SOX2 in NC, hMOF OVCAR3 cells (**F**) and sh-NC, sh-hMOF-1, sh-hMOF-2 A2780 cells (**G**). **H**, **I** Effects of hMOF on apoptosis were detected by flow cytometry. **J**, **K** Mitochondrial membrane potential was assessed by flow cytometry after TMRE staining. **L**, **M** Cells were cultured in medium containing different concentrations of cisplatin for 48 h, cell viability rates were respectively detected by MTT. Compared with NC group, *P < 0.05 and **P < 0.01 showed significantly difference. Compared with sh-NC group, ^#^*P* < 0.05 and ^##^*P* < 0.01 showed significantly difference.

Next, genetic overexpression or knockdown of hMOF in OC cells were to further investigate the effects of hMOF on OC stemness characteristics and cisplatin-resistance. The hMOF expressions in a panel of OC cell lines, OVCAR3, SKOV3, ES2, and A2780 were detected by western blot assay (Fig. 2A). Lentivirus mediated hMOF stable high expression OVCAR3 cells were constructed and identified, which named NC and hMOF cells (Fig. 2B). By the same method, we selected A2780 cells to construct hMOF stable low expression cell lines, named sh-NC, sh-hMOF-1, sh-hMOF-2 cells (Fig. 2C). To evaluate the effect of hMOF expression on the stemness characteristics of OC, the sphere formation assay was performed. hMOF overexpression OVCAR3 cells exhibited higher sphere-forming capacity (Fig. 2D) and higher expression levels of stemness related genes (Nanog, OCT4 and SOX2) than those of compared control cells (Fig. 2F). Conversely, knockdown of hMOF in A2780 cells significantly decreased the sphere-forming capacity and inhibited the expression of stemness related genes when compared to sh-NC cells (Fig. 2E, G). Besides, overexpression of hMOF led to an obvious decrease in cisplatin-induced cell apoptosis, mitochondrial damage, and loss of cell viability compared with NC cells (Fig. 2H, J, L). Inversely, compared to sh-NC cells, knockdown of hMOF resulted in much greater cell apoptosis rate, mitochondrial damage, and decreased cell viability when the cells were treated with cisplatin (Fig. 2I, K, M).

### hMOF increases the resistance to cisplatin treatment in vivo

**Fig. 3:**
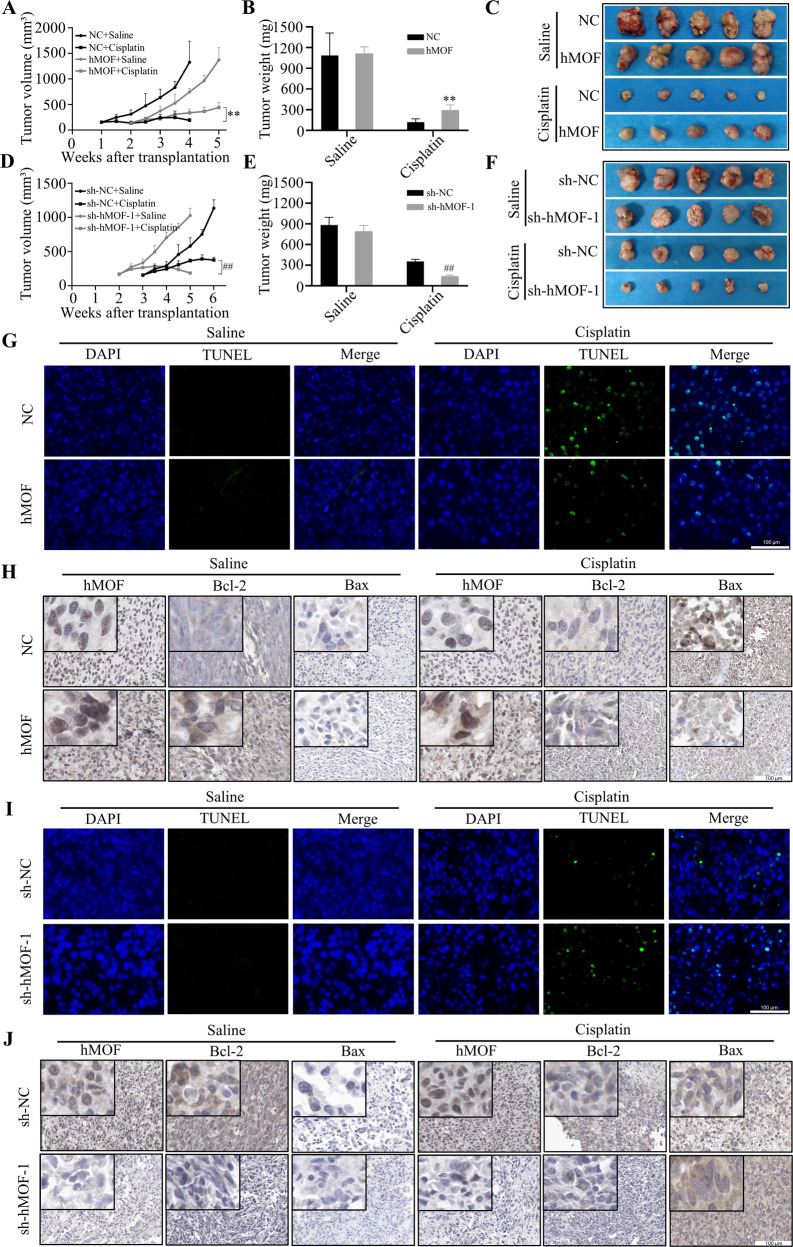
hMOF increases the resistance to cisplatin treatment in vivo. **A** The tumor growth curves, **B** Tumor weight and **C** tumor pictures of NC group and hMOF group after cisplatin or saline treatment. **D** The tumor growth curves, **E** tumor weight and **F** tumor pictures of sh-NC group and sh-hMOF-1 group after cisplatin or saline treatment. **G**, **I** The effects of hMOF on cell apoptosis in tumor tissues were monitored by TUNEL assay, and green fluorescence indicated TUNEL-positive staining. **H**, **J** Representative images of immunohistochemistry of hMOF, Bcl-2 and Bax in tumor tissues. Compared with NC group, **P < 0.01 showed significantly difference. Compared with sh-NC group, ^##^*P* < 0.01 showed significantly difference.

According to the above experimental data, we proved that high-expressed hMOF could promote cisplatin resistance in vitro OC cells. Thus, animal experiments were performed to further verify the role of hMOF on cisplatin resistance in vivo. Specifically, high-expressed hMOF did inhibit tumor growth rate, the growth time for tumor volume to reach about 150mm^3^ in two groups was one week for NC group and two weeks for hMOF group, respectively (Fig. 3A). After treated with saline or cisplatin for three weeks, the mice were euthanized and the tumor were weighed. At the end of cisplatin treatment, the tumor volume and weight in hMOF group were obviously larger than NC group (Fig. 3B, C). On the contrary, knockdown of hMOF accelerated the tumor growth rate (Fig. 3D) and resulted in better cisplatin sensitivity (Fig. 3E, F).

TUNEL assay was performed on the tumor tissues to detect apoptotic cells in different groups. After treated with cisplatin, more TUNEL-positive cells were observed in tumor tissues of NC group than that of hMOF group (Fig. 3G), and TUNEL-positive cells of tumor tissues were significantly increased in sh-hMOF-1 group compared with sh-NC group (Fig. 3I). Subsequently, the expressions of hMOF, Bcl-2 and Bax were detected by immunohistochemistry (IHC). High-expressed hMOF was associated with high expression of anti-apoptotic protein Bcl-2 and low expression of pro-apoptotic protein Bax (Fig. 3H, J). Therefore, we concluded that elevated hMOF inhibited cell apoptosis and enhanced resistance to cisplatin in vivo.

### Transcriptome sequencing results and validation

**Fig. 4:**
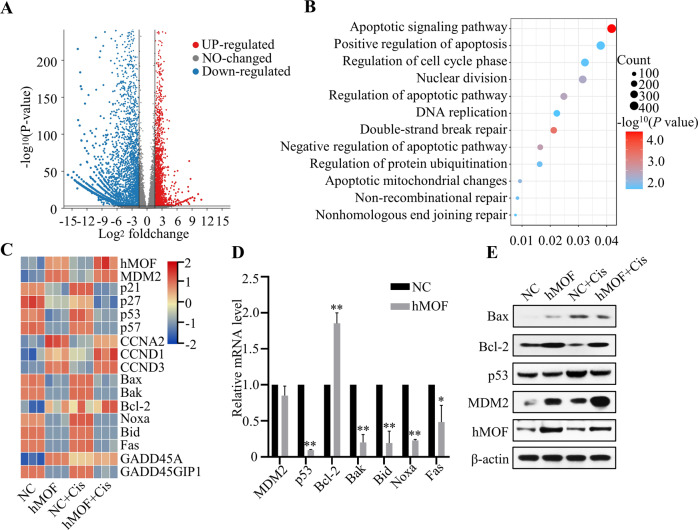
Transcriptome sequencing results and validation. **A** The volcano plot of transcriptome sequencing in OVCAR3 cells transfected with the hMOF gene and negative control. **B** Kyoto Encyclopedia of Genes and Genomes (KEGG) pathway enrichment analysis of differential genes. **C** Heatmap analysis of significantly altered genes. **D** The mRNA levels of MDM2, p53, Bcl-2, Bak, Bid, Noxa, and Fas were detected by qRT-PCR in NC cells and hMOF cells. **E** Western blot analysis of Bax, Bcl-2, p53, MDM2, and hMOF proteins expression before and after treatment with cisplatin (10 µmol/L, 24 h). Compared with NC group, *P < 0.05 and **P < 0.01 showed significantly difference.

To gain insight into the specific mechanism of hMOF promoting cisplatin resistance in OC cells, we performed RNA sequencing to identify the differentially expressed genes between NC cells and hMOF cells. The volcano plot graph in Fig. 4A indicated significant alterations of the RNAs expression when hMOF gene was overexpressed in OVCAR3 cells. KEGG pathway enrichment analysis was conducted to further study the differential genes. Apoptosis pathway was obviously enriched in addition to DNA repair, DNA replication, cell cycle regulation, and so on (Fig. 4B). Heatmap showed the relative expression of apoptosis relevant genes. Red represented higher expression and blue represented lower expression, compared to the mean value of the gene across all test samples. hMOF overexpression resulted in up-regulation of MDM2 and down-regulation of p53 (Fig. 4C). The qRT-PCR and western blot were used to further validate the RNA sequencing results. The mRNA expression of p53, Bcl-2, Bak, Bid, Noxa, and Fas showed the consistency with the RNA sequencing, while MDM2 mRNA did not change significantly (Fig. 4D). Then, as shown in Fig. 4E, hMOF markedly promoted MDM2 protein accumulation compared with NC cells regardless of whether OC cells were treated with cisplatin or not. The expressions of pro-apoptotic proteins Bax and p53 decreased, meanwhile, anti-apoptotic protein Bcl-2 was obviously increased in hMOF cells compared to NC cells when treated with cisplatin. These results further proved that high expression of hMOF could reduce the sensitivity of OC cells to cisplatin.

### hMOF improves the stability and expression of MDM2 protein through the ubiquitin-proteasome pathway

**Fig. 5:**
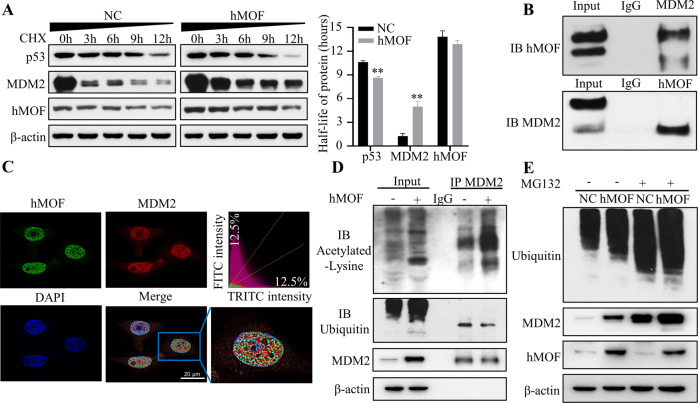
hMOF improves the stability and expression of MDM2 protein through the ubiquitin-proteasome pathway. **A** Cycloheximide (100 µg/mL) acted on cells at different time points, the degradation rate and half-life of hMOF, MDM2, and p53 proteins were detected by western blot. **B** Co-immunoprecipitation and immunoblotting for hMOF-MDM2. **C** Immunofluorescence images for hMOF (green) and MDM2 (red) in hMOF-overexpressed OVCAR3 cells. DAPI was used as a nuclear stain (blue). Colocalization of red and green fluorescence appeared yellow. Double fluorescence assays showing colocalization of hMOF and MDM2 (Pearson’s correlation = 0.669086). **D** Co-immunoprecipitation and immunoblotting analysis of the acetylation and ubiquitination levels of MDM2 protein. **E** Western blot was used to detect ubiquitination levels and MDM2 expression in the presence or absence of MG132 (20 µg/mL) in NC cells and hMOF cells. Compared with NC, **P < 0.01 showed significantly difference.

In the above studies, we found that overexpressed hMOF could significantly improve the expression of MDM2 protein, however, there was no significant alter in MDM2 mRNA level. Therefore, we speculated that hMOF could regulate the stability and expression of MDM2 at the post-translational level. The cycloheximide (CHX, 100 µg/mL), a protein biosynthesis inhibitor, was used to estimate the half-life of proteins. Overexpression of hMOF could dramatically reduce the degradation rate and prolong the half-life of MDM2 protein. Correspondingly, the degradation rate of p53 protein was increased in hMOF cells, and the half-life of hMOF protein did not alter significantly (Fig. 5A). hMOF could obviously improve the stability and expression of MDM2 protein, further, we speculated that there may be a physical interaction between hMOF and MDM2. To test our conjecture, MDM2 protein was immunoprecipitated, and immunoblotting was used to detect the expression of hMOF. The result revealed that MDM2 structurally interacted with hMOF and vice versa (Fig. 5B). Double-labeling immunofluorescence further showed the co-localization of hMOF and MDM2 mainly in the nucleus (Fig. 5C).

The experimental results showed that hMOF directly interacted with MDM2. Since hMOF is a histone acetylating transferase, we next explored whether MDM2 protein is a non-histone substrate of hMOF. As a way of testing the above speculation, we detected the acetylation of MDM2 protein and found that hMOF could promote the acetylation of MDM2. Previous research revealed that acetyltransferase p300 acetylated the MDM2, MDM2 acetylation blocked the MDM2 self-ubiquitination and stabilized MDM2 protein [32]. Next, the ubiquitylation and acetylation of MDM2 were detected by Co-IP analysis, the research results showed that high-expressed hMOF led to an increase in the acetylation levels and a decrease in the ubiquitylation levels of MDM2 protein. Meanwhile, hMOF could promote MDM2 accumulation and enhance the overall acetylation levels in cells (Fig. 5D). To further verify the stability and expression of MDM2 regulated by hMOF through ubiquitin-proteasome pathway, proteasome inhibitor MG132 (20 µg/mL) was added in cell culture medium. The hMOF-mediated MDM2 accumulation effects were partially counteracted (Fig. 5E). Based on the above experimental results, we concluded that hMOF interacted with MDM2, and MDM2 protein was a new non-histone substrate of hMOF. Taken together, overexpressed-hMOF could promote the acetylation and inhibit the ubiquitination of MDM2 protein, and ultimately stabilize MDM2 protein through the ubiquitin-proteasome pathway.

### Downregulation of MDM2 can reverse hMOF-induced cisplatin-resistance

**Fig. 6:**
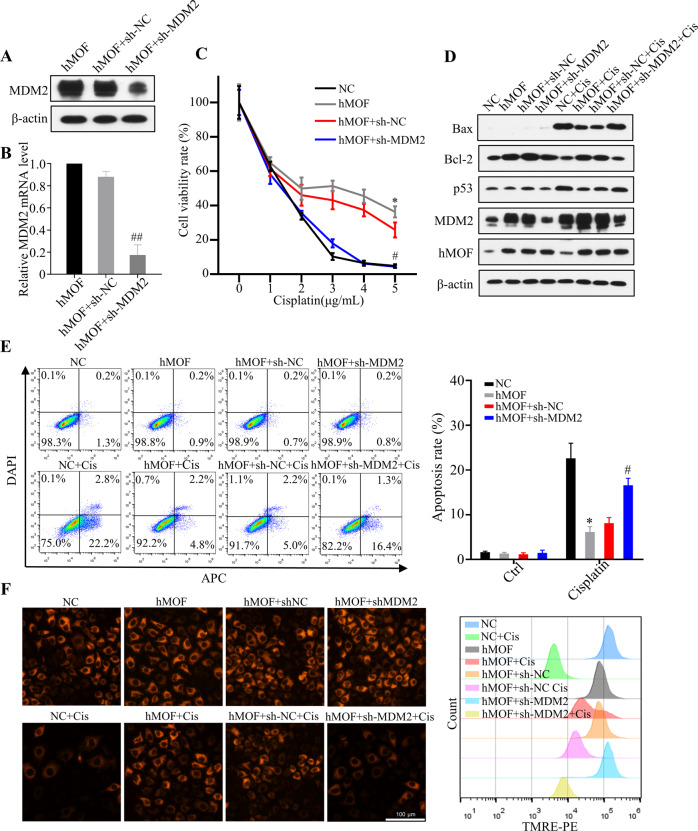
Downregulation of MDM2 can reverse hMOF-induced cisplatin-resistance. **A** Overexpressed hMOF cells (hMOF cells) were transfected with control shRNA (named hMOF+sh-NC) or shRNA targeting MDM2 (named hMOF+sh-MDM2), and the MDM2 protein expression (**A**) and mRNA level (**B**) were analyzed by western blot and qRT-PCR. **C** Cells were treated with indicated concentrations of cisplatin for 48 h, cell viability rate was monitored by MTT. **D** The expressions of Bax, Bcl-2, p53, MDM2 and hMOF were detected by western blot in the presence or absence of cisplatin in cells. The impact for knockdown of MDM2 gene in hMOF cells on apoptosis (**E**) and mitochondrial membrane potential (**F**) were detected by flow cytometry. Compared with NC, *P < 0.05 showed significantly difference. Compared with hMOF+sh-NC, ^#^*P* < 0.05 and ^##^*P* < 0.01 showed significantly difference.

In order to verify whether the effects of hMOF on cisplatin resistance depending on MDM2, hMOF cells were transfected with a specific shRNA targeting MDM2. Efficient downregulation of MDM2 levels was detected by WB and qRT-PCR (Fig. 6A, B). After treated with indicated concentrations of cisplatin for 48 h, hMOF+sh-MDM2 cells showed a lower cell viability compared with hMOF+sh-NC cells or hMOF cells. The results of MTT assay indicated that the enhanced cisplatin resistance caused by high-expressed hMOF could be reversed by interfering with MDM2 (Fig. 6C). As shown in Fig. 6D, after cisplatin treatment, the expression of pro-apoptotic proteins Bax and p53 were markedly increased in hMOF+sh-MDM2 cells compared with hMOF+sh-NC cells, while anti-apoptotic protein Bcl-2 was obviously decreased in hMOF+sh-MDM2 cells. Flow cytometry results further showed the significant increase both in apoptosis rate and mitochondrial damage when MDM2 expression was interfered in hMOF cells (Fig. 6E, F). The detections of apoptosis related proteins and flow cytometry assay showed that the inhibitory effect of hMOF on cisplatin-mediated apoptosis could be reversed when MDM2 expression was disturbed. These above experimental data demonstrated that MDM2 was essential for the effect of hMOF on cisplatin resistance to OC cells.

### Knockdown of hMOF can improve the sensitivity of OVCAR3/DDP cells to cisplatin in vivo tumor model

**Fig. 7:**
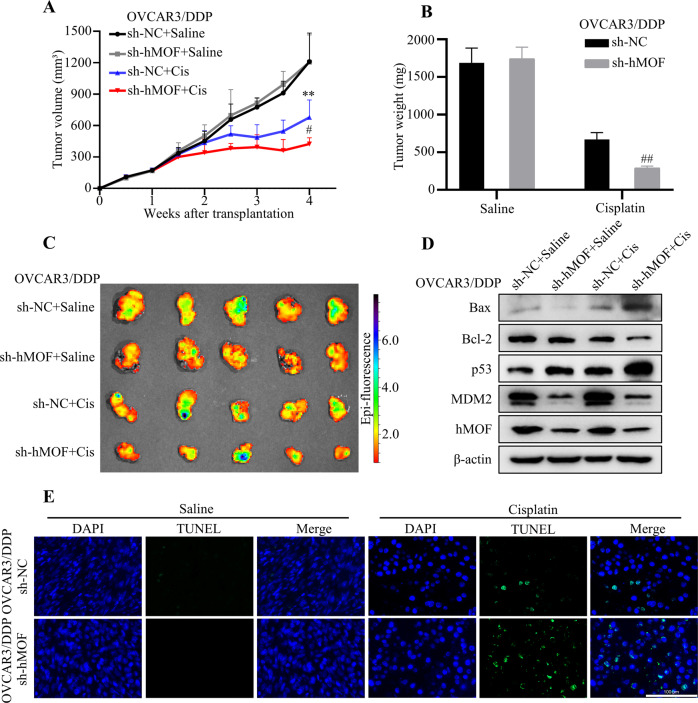
Knockdown of hMOF can improve the sensitivity of OVCAR3/DDP cells to cisplatin in vivo. Adenovirus shRNA targeting hMOF was used to knockdown the expression of hMOF in OVCAR3/DDP cells. The tumor growth curve (**A**), tumor weight (**B**), and tumor fluorescence image (**C**) of OVCAR3/DDP cells after being treated with saline or cisplatin in sh-NC and sh-hMOF groups. **D** Western blot analysis of Bax, Bcl-2, p53, MDM2, and hMOF proteins expression treated with saline or cisplatin in sh-NC and sh-hMOF groups. **E** TUNEL staining was used to detect the apoptotic cells in sh-NC and sh-hMOF groups treated with saline or cisplatin. Compared with sh-NC+Saline, **P < 0.01 showed significantly difference. Compared with sh-NC+Cis, ^#^ *P* < 0.05 and ^##^ *P* < 0.01 showed significantly difference.

The animal studies were conducted to verify whether adenovirus interference with hMOF expression can reverse cisplatin resistance in ovarian cancer OVCAR3/DDP cells in vivo. After tumor formation in xenografted mice, the adenoviruses with negative control (sh-NC) or targeting hMOF (sh-hMOF) were respectively injected into the tumor, and then the mice were treated intraperitoneally with saline or cisplatin. As shown in Fig. 7A–C, the tumor volume and weight in sh-hMOF+Cis group were significantly reduced compared with sh-NC+Cis group, indicating adenovirus that interfering with hMOF expression in combination with cisplatin could increase the sensitivity of tumor tissues to cisplatin and reverse cisplatin resistance. Further, the expressions of Bcl-2, Bax, p53, hMOF and MDM2 were analyzed by western blot. The expressions of hMOF and MDM2 were reduced markedly both in sh-hMOF+Saline and sh-hMOF+Cis groups, which proved the effectiveness of adenovirus interference. Meanwhile, the expressions of Bax and p53 were apparently higher in sh-hMOF+Cis group than those in sh-NC+Cis group. On the contrary, anti-apoptotic protein Bcl-2 was significantly reduced in sh-hMOF+Cis group compared with sh-NC+Cis group (Fig. 7D). A greater percentage of TUNEL-positive cells were observed in sh-hMOF tumor tissue sections after cisplatin treatment (Fig. 7E). These results suggested that knockdown of hMOF can improve the sensitivity of OVCAR3/DDP cells to cisplatin and reverse the cisplatin resistance in *vivo* tumor model.

## Discussion

Ovarian cancer is known to the highest mortality rate among all the gynecologic tumor [1]. Cisplatin (DDP) is the first platinum-based compound approved by the Food and Drug Administration (FDA) to treat OC patients [33]. Cisplatin binds to DNA, induces DNA damage, inhibits DNA replication, ultimately leads to apoptosis or programmed cell death [34]. Cisplatin resistance is a major obstacle to the treatment of OC in clinical practice. Hence, it is crucial to explore the mechanisms of cisplatin resistance in the treatment of OC patients. hMOF is the major histone H4 lysine 16 acetyltransferase in mammals and contributes to regulating the gene transcription, cell cycle progression and chromatin structure maintenance by acetylation modification of histones [35]. However, the role of hMOF on cisplatin resistance in OC and the relevant mechanisms remain unclear. In this study, we found that hMOF may inhibit cell apoptosis signaling pathway by analyzing gene expression data from TCGA database. Next, we analyzed the correlation between hMOF expression and cisplatin sensitivity by GDSC database and discovered that hMOF showed a positive correlation with cisplatin IC_50_ value. Previous study had shown that MOF played an essential role in the maintenance of embryonic stem cells (ESCs) self-renewal and pluripotency [13]. Cancer stem cells (CSCs) are a subset of tumor cells that are capable of self-renewal, differentiation, and tumorigenesis. CSCs can evade multiple drug actions with the aid of multiple intrinsic and external mechanisms including DNA damage repair pathway activation, high-level expression of drug efflux-related proteins, the influence of epithelial-to-mesenchymal transition (EMT), abnormal angiogenesis, and so on [36–38]. Consistent with previous studies, in this research, the volume of the formed spheres, sphere-forming capacity, and stemness-related genes expression were significantly enhanced in the cisplatin-resistant OVCAR3/DDP cells compared to OVCAR3 cells. Furthermore, our data suggested that high-expressed hMOF could promote the stemness characteristics, markedly inhibit the apoptosis and reduce the sensitivity of OC cells to cisplatin in vivo *and* in vitro *e*xperiments.

As a histone acetyltransferase, hMOF can catalyze the acetylation of histone H4 lysine 16 and loss of MOF can lead to defects in ionizing radiation-induced DNA damage repair [39]. Besides, MOF played important roles in multiple steps in the DNA damage response and double-strand break repair [40]. p53 was a non-histone substrate of hMOF and apoptosis induction was the central anticancer function of p53 protein. hMOF induced acetylation of p53 at lysine 120 after DNA damage to activate pro-apoptotic target genes Bax and PUMA [41]. hMOF dynamically interacted with and activated Nrf2 through acetylation at lysine 588. hMOF knockdown could enhance CDDP, 5-FU and bleomycin-induced decrease of survival of NSCLC cells by regulating Nrf2 dependent genes [14]. These studies indicated that hMOF could regulate DNA damage repair and cell apoptosis by acetylating both histone and non-histone proteins. RNA sequencing analysis revealed that MDM2 and downstream apoptosis-related genes showed significant changes, and subsequent experiments confirmed that hMOF could induce accumulation of MDM2 protein.

Cisplatin-mediated the irreparable damage and apoptosis depend on stability and activity of p53 protein [42]. MDM2 is a key negative regulator of p53, binds to p53 and functions as an E3 ubiquitin ligase to promote the ubiquitination and degradation of p53 via the proteasome [43]. Targeting MDM2 has recently attracted interest in cancer chemotherapy resistance due to its potential to restore tumor suppressor function of p53. In the research of multiple myeloma, targeting an MDM2/MYC axis can trigger remarkable apoptosis and overcome drug resistance [44]. Another study reveals that genetic or chemical inhibition of MDM2 obviously blocked Y-box-binding protein-1-mediated temozolomide resistance in glioma cells [45]. A novel inhibitor of MDM2 (termed SP-141) exerts anti-breast cancer activity in vitro *and* in vivo via directly binding to MDM2 and promoting MDM2 proteasomal degradation [46]. MDM2 is an unstable protein and regulated by post-translational modifications, such as phosphorylation, ubiquitination and acetylation. The E3 ubiquitin ligase RNF12 targets MDM2 for ubiquitination and proteasomal-dependent degradation. RNF12 elevates p53 protein level though abrogating MDM2-mediated p53 degradation, finally promotes DNA damage-induce apoptosis in a p53 dependent manner [47]. Ubiquitination and acetylation are mutually exclusive modifications, and the competition between these two modifications can affect the stability of MDM2 protein. Acetyltransferase p300 acetylated MDM2, and MDM2 acetylation could block the self-ubiquitination of MDM2. MDM2 acetylation triggered p53 ubiquitination and reduced the stability of p53 protein, inhibited cellular apoptosis in response to DNA damage [27].

In our study, we confirmed that hMOF could directly interact with MDM2, promote MDM2 accumulation by inhibiting its ubiquitinated degradation. hMOF significantly prolonged the half-life of MDM2 protein and shortened the half-life of p53. Furthermore, the cisplatin resistance induced by hMOF was dependent on MDM2 expression. Knockdown of hMOF could enhance the sensitivity of OVCAR3/DDP cells to cisplatin and reverse the cisplatin resistance in vivo.

## Conclusion

Collectively, our research demonstrated that MDM2 interacted with hMOF, MDM2 was a new non-histone substrate of hMOF. hMOF reduced cisplatin-induced p53 accumulation by stabilizing MDM2 expression. hMOF/MDM2 axis might be a potential target for the treatment of chemotherapy-resistant OC.

## Methods

### Data sets and networks

RNA-sequencing expression profiles and corresponding clinical information for 376 ovarian serous cystadenocarcinoma were downloaded from the TCGA (The Cancer Genome Atlas) dataset (https://portal.gdc.cancer.gov/). R GSVA package with the parameter method = ‘ssgsea’ was used to analyze. The correlation between hMOF and apoptosis pathway scores was analyzed by Spearman correlation. The hMOF and stemness-related genes correlation map was realized by the R software package “ggstatsplot”. The chemotherapeutic response to cisplatin was predicted based on the largest available public pharmacogenomics database GDSC [https://www.cancerrxgene.org/]. The prediction process was implemented by R package “pRRophetic”. All parameters were set as the default values. All the analysis methods and R packages were implemented by R version 4.0.3.

### Cell culture

The human ovarian cancer cell lines of OVCAR3, SKOV3, ES2, and A2780 were obtained from the cell bank of the Chinese Academy of Sciences (Shanghai, China). The OVCAR3 and A2780 cells were cultured in RPMI 1640 Medium (C3010-0500, Biological Industries, Israel). The SKOV3 and ES2 cells were cultured in McCoy’s 5 A Medium (C3020-0500, Biological Industries, Israel). Growth medium was supplemented with 10% fetal bovine serum (C04001-500, Biological Industries, Israel), 100 U/mL penicillin and 100 µg/mL streptomycin. Cells were incubated in a humidified environment of 37 °C and 5% CO_2_.

### MTT assay

Cells in the logarithmic growth phase were seeded in 96-well plates at a density of 1 × 10^3^ cells/well. After 24 h, the cells were treated with 200 µL cell culture medium containing various concentrations of cisplatin (0, 1, 2, 3, 4, 5 µg/mL) for 48 h. After that, 20 µL of 5 mg/mL MTT solution were added to each well and incubated for another 4 h. The supernatant was discarded and 150 µL DMSO was added to each well. The absorbance was measured by a Spark microplate reader (Tecan, orf, Switzerland) at 490 nm.

### OVCAR3/DDP cell line establishment

The cisplatin resistant cell line OVCAR3/DDP were established with gradually increased concentration of cisplatin (#HY-17394, MCE, Shanghai, China) from 2.5 µM to 20 µM over a period of 6 months as previously described [32]. Briefly, OVCAR3 cells were incubated with 2.5 µM cisplatin for 3 days and recovery for the following 3 days. After repeating 2 cycles at 2.5 μM cisplatin concentration, the cells were then treated with 5 μM cisplatin in the following 2 cycles. This procedure was continued with increasing cisplatin concentration up to 20 μM. During the cisplatin-resistance induction procedure, the IC_50_ values of every 3 passage cells were accessed in comparison with those of the parental cells until the IC_50_ value remained constant. The cisplatin-resistant cell lines obtained by this method were maintained in growth media containing 10 μM cisplatin. To verify the effectiveness of OVCAR cells resistant to cisplatin, the inhibitory concentration (IC_50_) value was determined using MTT. Inhibition (%) = {1 − [OVCAR3/DDP optical density (OD) − Blank OD]/(OVCAR3 OD − Blank OD)} × 100%. Resistance index = OVCAR3/DDP half-maximal inhibitory concentration (IC_50_)/OVCAR3 IC_50_.

### Sphere-forming assay

Sphere-formation assay is the gold standard for evaluating cancer stem cells (CSCs). It judges the ability of a single cell to self-renew under suitable medium conditions. In general, it is necessary to test the self-renewal ability of cells after successive passage. Single-cell suspensions (1 × 10^3^ cancer cells/ well) were seeded in a poly-HEMA coated 6-well plate in MammoCult^TM^ medium (#05620, STEMCELL Technologies, Vancouver, Canada). After 10 days, tumor spheres were filtered by 70 µm cell filter (BD), then washed with PBS, and then centrifuged at 1000 rpm for 5 min to collect tumor spheres, and digested into single cells by 0.25% pancreatin. 1 × 10^3^ cells were collected for secondary pellet culture. Tumor spheres with a diameter larger than 75 µm were counted under an invert microscopy and sphere colony formation efficiency (SFE) was evaluated according to the following formula: (numbers of colonies/numbers of cells inoculated) × 100%.

### Western blot

Total protein was extracted from treated cells and tumor tissues using RIPA buffer supplemented with 1×protease inhibitor cocktail (#HY-K0010, MCE, Shanghai, China) and phosphatase inhibitors (#HY-K0022, MCE), and the protein concentration was qualified by BCA kit (#23225, Thermo Scientific, Waltham, USA). Protein samples were separated by 10% SDS-PAGE and then transferred onto nitrocellulose membranes. Membranes were treated with 5% skim milk for 2 h at room temperature and incubated with primary antibodies overnight at 4 °C. After washed with PBST buffer, the membranes were soaked in corresponding horse-radish peroxidase (HRP)-labeled secondary antibodies for 2 h at room temperature. The antibodies used were as follows: hMOF (#ab200660, 1:1000, Abcam, Cambridge, UK), MDR1 (#ab170904, 1:1000, CST, Boston, Massachusetts, USA), MRP1 (#72202, 1:1000, CST), ABCG2 (#42078, 1:1000, CST), Nanog (#14295-1-AP, 1:1000, Proteintech, Wuhan, China), OCT4 (#11263-1-AP, 1:1000, Proteintech), SOX2 (#ab92494, 1:1000, Abcam), MDM2 (#ab259265, 1:1000, Abcam), p53 (#ab26, 0.1 μg/ml, Abcam, USA), β-actin (#TA-09, 1:1000, Zhongshan Golden Bridge, Beijing, China), Bax (#ab32503, 1:1000, Abcam, USA), Bcl-2 (#ab32124, 1:1000, Abcam), Acetylated-lysine (#9441S, 1:1000, CST), ubiquitin (#382766, 1:1000, ZENBIO, Wuhan, China), goat anti-rabbit IgG (#ab6721, 1:5000, Abcam), goat anti-mouse IgG (#ab205719, 1:5000, Abcam). The protein immune complexes were detected by ECL (#34580, Thermo Scientific).

### Quantitative real-time PCR (qRT-PCR)

Total RNA was isolated by Trizol reagent (15596026, Invitrogen, California, USA) and cDNA was synthesized using a reverse transcription kit (R333, Vazyme Biotech Co., Ltd, Jiangsu, China). RNA quantitation was conducted by ChamQ Universal SYBR® qPCR Master Mix kit (Q711, Vazyme Biotech Co., Ltd, Jiangsu, China) in StepOnePlus™ Real-Time PCR System (Applied Biosystems, USA) and the data was analyzed using the 2^-∆∆Ct^ relative expression quantity. The specific primers synthesized by Sangon Biotech Co., Ltd were listed in Supplementary Table. 1.

### Cell transfection

The recombinant lentiviruses were constructed by Shanghai Genechem Co., LTD. The low hMOF expression cell line OVCAR3 and high hMOF expression cell line A2780 were plated at a density of 1 × 10^5^ cells/well in 6-well plates and transfected with lentiviruses (MOI = 50). The empty lentiviral vector was taken as a negative control. After transfection with lentiviruses for 48 h, the positive cells were selected by puromycin for two weeks, and monoclonal cells were amplified to prepare stably transfected cell lines for the follow-up experiments. MDM2-specific RNAi plasmids (TACCTACTGATGGTGCTGTAA) with neomycin resistance and control RNAi plasmids were also designed by Genechem Co., LTD and used to interfere with MDM2 expression in hMOF overexpressed cells. The sequences of shRNA were as follows: (shRNA-hMOF-1: cgAAATTGATGCCTGGTATTT; shRNA-hMOF-2: gcAAGATCACTCGCAACCAAA).

### Cell apoptosis

After treated with cisplatin (10 µmol/L) for 24 h, all the cells were collected and washed 3 times with cold PBS. Then the cells were stained with Annexin V-APC/DAPI double staining solution (KGA1021, KeyGen Biotech, Jiangsu, China) for 20 min in the dark at room temperature. The apoptosis cells were detected by ACEA NovoCyte Flow cytometer (ACEA Biosciences, Hangzhou, China) and the data were analyzed with FlowJo software (FlowJo, Ashland, OR, USA).

### Mitochondrial membrane potential examination

Mitochondrial membrane potential (MMP), a key indicator of mitochondrial function, was assessed by the fluorescence probe tetramethylrhodamine ethyl ester TMRE assay system (C2001S, Beyotime Biotechnology, Shanghai, China). After treated with cisplatin (10 µmol/L) for 24 h, all the cells were washed 3 times with PBS and incubated with TMRE working solution for 20 min at 37 °C. The MMP could be assessed through TMRE fluorescence changes which visualized using Lecia DMi8 fluorescence microscopy (Leica, Germany) and quantitatively analyzed by flow cytometry (ACEA Biosciences, Hangzhou, China).

### Animal experiments

The animal experiments were performed in accordance with the guidelines of the Institutional Animal Care and all the animal procedures were approved by the Anima Ethics Committee of the Laboratory Animal Center of Zhengzhou University. 4–6 weeks old NOD-SCID female mice were purchased from Beijing Sibeifu Bioscience Co., Ltd (Beijing, China). 0.1 mL serum-free cell culture medium containing 5 × 10^6^ cells were subcutaneously inoculated in the right axilla of mice. After transplantation, the size of the tumor was measured twice a week. Five mice per group were randomly assigned and when the tumor volume reached 150 mm^3^, the xenografted mice were treated intraperitoneally with saline or 5 mg/Kg cisplatin twice a week for 2 weeks. Besides, adenovirus expressing shRNA of hMOF or negative control were intratumorally injected (5 × 10^8^ PFU per mouse) in OVCAR3/DDP xenografted mice before initial intraperitoneal injection of saline or cisplatin. All the measurements and treatments were done by blinded investigators. After the 21st day of treatment, all the mice were euthanized and the tumors were collected for following experiments. Tumors were weighed and the fluorescence of tumors in OVCAR3/DDP xenografted mice were evaluated by an optical imaging system (PerkinElmer, IVIS Spectrum In Vivo Imaging System, American).

### Immunohistochemistry (IHC) assay

A total of 30 paraffin-embedded OC samples were collected from patients who underwent surgical resection at the Department of Gynecology of the First Affiliated Hospital of Zhengzhou University. The patient information was anonymized and de-identified before its analysis. A pathological diagnosis of primary OC was confirmed in all samples, and six of the cases who receiving platinum-based chemotherapy were considered to have platinum-resistant disease or persistent disease. The IHC assay was performed by the SPlink Detection Kits (SP-9000, Zhongshan Golden Bridge Biotechnology, Co., Ltd., China) according to the manufacturer’s instructions. Briefly, the paraffin slides were deparaffinized with xylene, rehydrated by a graded of alcohol, and conducted antigen repair in boiled sodium citrate buffer (pH 6.0). After that, the sections were immersed in endogenous peroxidase blocking agent and sealed with goat serum at room temperature. The tissue sections were incubated with corresponding primary antibodies at 4 °C overnight. The primary antibodies used were as follows: hMOF (#ab200660, 1:500, Abcam), Bcl-2 (#ab32124, 1:250, Abcam), Bax (#ab32503, 1:250, Abcam). On the following day, the sections were incubated with HRP-conjugated goat anti-rabbit secondary antibody. Finally, the intensity of detected protein was visualized by DAB solution and the nuclei were stained with hematoxylin.

### TUNEL assay

The apoptosis in situ of tissue sections was detected by TUNEL BrightGreen Apoptosis Detection Kit (A112-01, Vazyme Medical Technology Co., Ltd., Jiangsu, China) according to the manufacturer’s protocol. After dewaxing and rehydration, the tissue sections were incubated with 20 µg/mL protease K solution at room temperature for 20 min. Then the tissues were equilibrated for 15 min and treated with 50 µL TdT incubation buffer for 1 h at 37 °C. Finally, the nuclei were stained with 2 µg/mL DAPI for 5 min in the dark. The positive signals were immediately recorded by a fluorescence microscopy under an excitation at 488 nm and an emission at 520 nm.

### RNA‑seq assay

The RNA-seq assay was performed by Annoroad (Beijing, China). Briefly, the total RNA of OVCAR3 cells was extracted by Trizol regent (Invitrogen life technologies, Carlsbad, CA, USA) and purified for library preparation and sequencing on an Illumina Hiseq platform. Preliminary screening was conducted on the original sequencing data based on the quality of the sequences. For data analysis, differential expression analysis of the two groups was performed using the DESeq2 R package (1.20.0), and genes were considered significantly differentially expressed when the p-value was less than 0.01 and |FoldChange| > 2. The Gene Ontology (GO) analysis was conducted on the GO database (http://www.geneontology.org/). Kyoto Encyclopedia of Genes and Genomes (KEGG) analysis was performed using KEGG Mapper (http://www.genome.jp/kegg/mapper.html). Enrichment analysis was performed using the R package clusterProfiler. Data visualization was performed with TBtools [48].

### Immunofluorescence analysis

hMOF-overexpressed OVCAR3 cells were seeded into plates containing glass coverslips and cultured for 24 h. The cell climbing slice was fixed with 4% paraformaldehyde for 2 h, permeabilized in 0.5% PBST (PBS containing 0.5% Triton X-100) for 10 min and blocked with 5% goat serum for 60 min. Then the cell slides were incubated with primary antibodies of hMOF (#ab200660, 1:1000, Abcam) and MDM2 (#66511-1-Ig, 1:500, Proteintech) at 4 °C overnight. After incubation with secondary antibodies (Alexa fluor 488 goat anti-rabbit, A0423, Beyotime, 1:1000; Alexa fluor 555 donkey anti-mouse, A0460, Beyotime, 1:1000) for 2 h at room temperature, the nuclei were counterstained with DAPI (1 μg/mL) for 5 min. The immunofluorescence images were captured by a Nikon NiE-A1 plus or Nikon C2+ confocal microscope, and analyzed using Nikon-elements AR software.

### Co-immunoprecipitation (Co-IP) experiments

The total protein extracted OVCAR3 cells was used to perform Co-immunoprecipitation (Co-IP) experiments. Appropriate proportion of primary antibodies were added to the protein lysate, and the antigen and antibodies were fully combined to form a complex by gentle rotating at 4 °C overnight. The MCE Protein A/G Magnetic Beads were added to the lysate then incubated at 4 °C for 2 h. The protein-conjugated magnetic Beads were collected and washed 5 times in lysis buffer using a magnetic rack. The protein complexes were mixed with SDS loading buffer and then used for western blot analysis.

### Statistical analysis

GraphPad Prism 8.0.2 (GraphPad Software, USA) was used to analyze the experimental data. In all experiments, triplicates were performed. Independent-sample and paired-sample *t*-tests were used to compare the differences between the two groups. One-way ANOVA analysis was used to compare more than two groups. *P* values less than 0.05 were statistically significant.

## Supplementary information

original image for blots

supplementary table.1

## Data Availability

Authors confirm the availability of data and materials in the study.
