# Supplementary figures and images for: hMOF induces cisplatin resistance of ovarian cancer by regulating the stability and expression of MDM2
Source: Cell Death Discov. 2023 Jun 8;9:179. doi: 10.1038/s41420-023-01478-y (PMC10250293; doi:10.1038/s41420-023-01478-y)

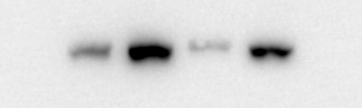

Supplement: Supplementary file 1 — original image for blots [file 41420_2023_1478_MOESM1_ESM.zip › original image for blots/original data files Figure 1F ABCG2.tif]

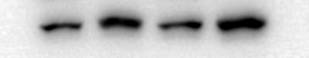

Supplement: Supplementary file 1 — original image for blots [file 41420_2023_1478_MOESM1_ESM.zip › original image for blots/original data files Figure 1F hMOF.tif]

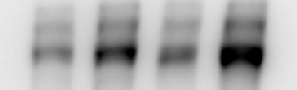

Supplement: Supplementary file 1 — original image for blots [file 41420_2023_1478_MOESM1_ESM.zip › original image for blots/original data files Figure 1F MDR1.tif]

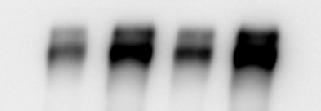

Supplement: Supplementary file 1 — original image for blots [file 41420_2023_1478_MOESM1_ESM.zip › original image for blots/original data files Figure 1F MRP1.tif]

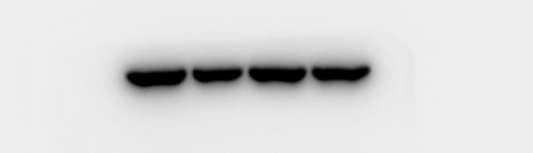

Supplement: Supplementary file 1 — original image for blots [file 41420_2023_1478_MOESM1_ESM.zip › original image for blots/original data files Figure 1F a┬-actin.tif]

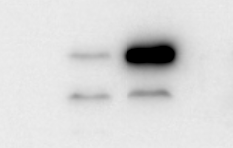

Supplement: Supplementary file 1 — original image for blots [file 41420_2023_1478_MOESM1_ESM.zip › original image for blots/original data files Figure 1I Nanog.tif]

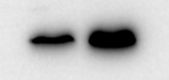

Supplement: Supplementary file 1 — original image for blots [file 41420_2023_1478_MOESM1_ESM.zip › original image for blots/original data files Figure 1I Oct4.tif]

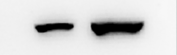

Supplement: Supplementary file 1 — original image for blots [file 41420_2023_1478_MOESM1_ESM.zip › original image for blots/original data files Figure 1I Sox2.tif]

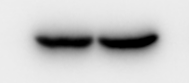

Supplement: Supplementary file 1 — original image for blots [file 41420_2023_1478_MOESM1_ESM.zip › original image for blots/original data files Figure 1I a┬-actin.tif]

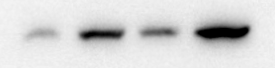

Supplement: Supplementary file 1 — original image for blots [file 41420_2023_1478_MOESM1_ESM.zip › original image for blots/original data files Figure 2A hMOF.tif]

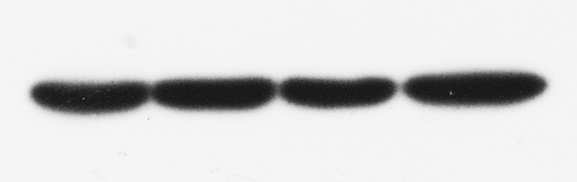

Supplement: Supplementary file 1 — original image for blots [file 41420_2023_1478_MOESM1_ESM.zip › original image for blots/original data files Figure 2A a┬-actin.tif]

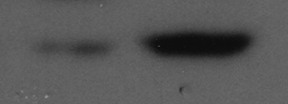

Supplement: Supplementary file 1 — original image for blots [file 41420_2023_1478_MOESM1_ESM.zip › original image for blots/original data files Figure 2B hMOF.tif]

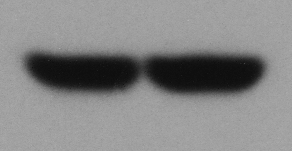

Supplement: Supplementary file 1 — original image for blots [file 41420_2023_1478_MOESM1_ESM.zip › original image for blots/original data files Figure 2B a┬-actin.tif]

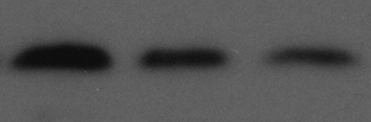

Supplement: Supplementary file 1 — original image for blots [file 41420_2023_1478_MOESM1_ESM.zip › original image for blots/original data files Figure 2C hMOF.tif]

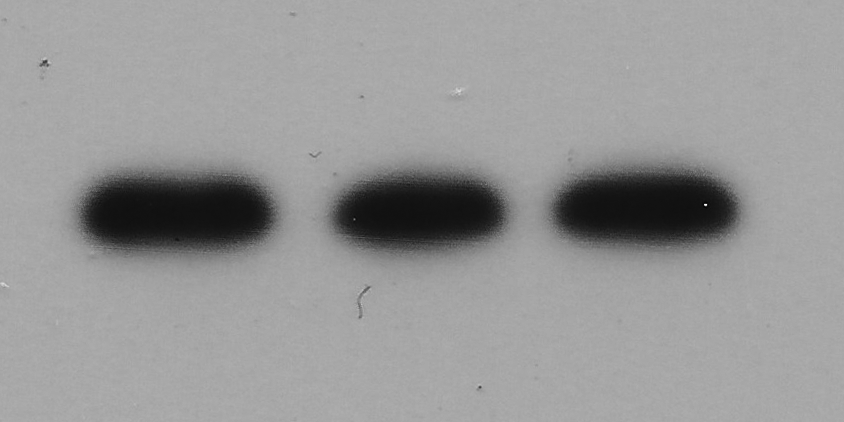

Supplement: Supplementary file 1 — original image for blots [file 41420_2023_1478_MOESM1_ESM.zip › original image for blots/original data files Figure 2C a┬-actin.tif]

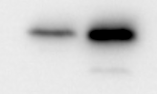

Supplement: Supplementary file 1 — original image for blots [file 41420_2023_1478_MOESM1_ESM.zip › original image for blots/original data files Figure 2F Nanog.tif]

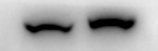

Supplement: Supplementary file 1 — original image for blots [file 41420_2023_1478_MOESM1_ESM.zip › original image for blots/original data files Figure 2F OCT4.tif]

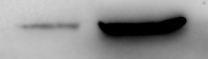

Supplement: Supplementary file 1 — original image for blots [file 41420_2023_1478_MOESM1_ESM.zip › original image for blots/original data files Figure 2F SOX2.tif]

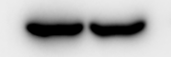

Supplement: Supplementary file 1 — original image for blots [file 41420_2023_1478_MOESM1_ESM.zip › original image for blots/original data files Figure 2F a┬-actin.tif]

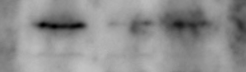

Supplement: Supplementary file 1 — original image for blots [file 41420_2023_1478_MOESM1_ESM.zip › original image for blots/original data files Figure 2G Nanog.tif]

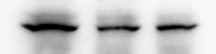

Supplement: Supplementary file 1 — original image for blots [file 41420_2023_1478_MOESM1_ESM.zip › original image for blots/original data files Figure 2G OCT4.tif]

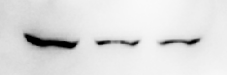

Supplement: Supplementary file 1 — original image for blots [file 41420_2023_1478_MOESM1_ESM.zip › original image for blots/original data files Figure 2G SOX2.tif]

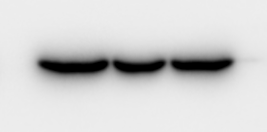

Supplement: Supplementary file 1 — original image for blots [file 41420_2023_1478_MOESM1_ESM.zip › original image for blots/original data files Figure 2G a┬-actin.tif]

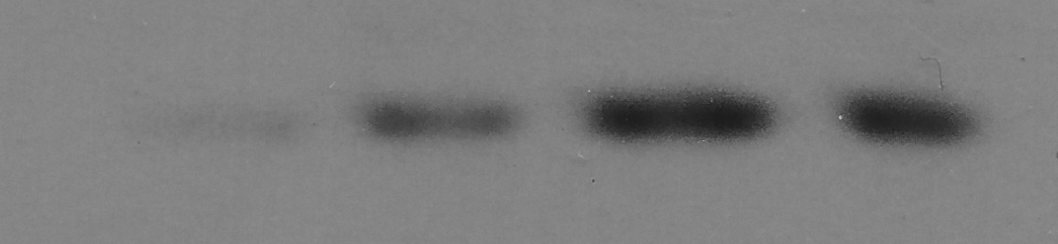

Supplement: Supplementary file 1 — original image for blots [file 41420_2023_1478_MOESM1_ESM.zip › original image for blots/original data files Figure 4E Bax.tif]

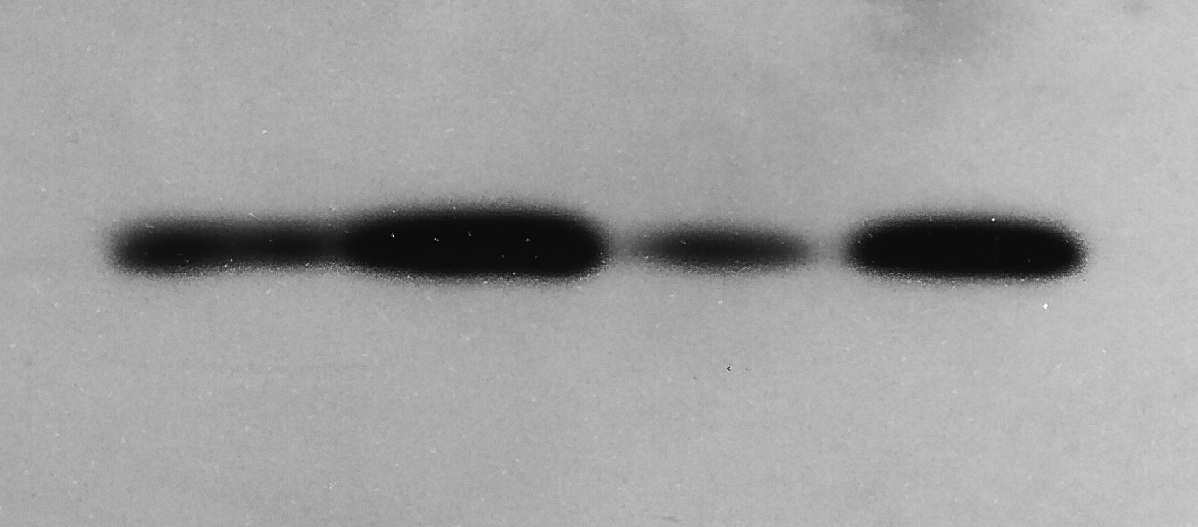

Supplement: Supplementary file 1 — original image for blots [file 41420_2023_1478_MOESM1_ESM.zip › original image for blots/original data files Figure 4E Bcl-2.tif]

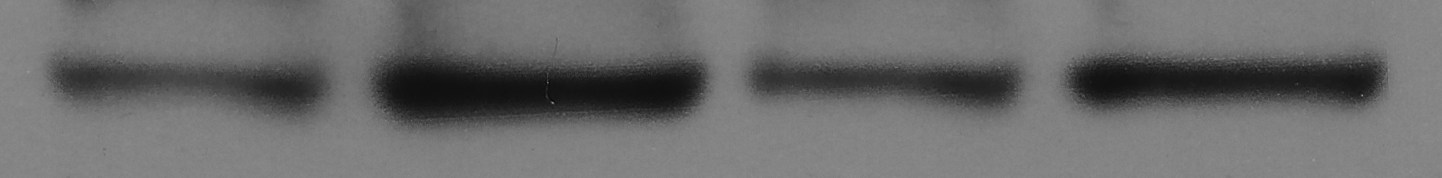

Supplement: Supplementary file 1 — original image for blots [file 41420_2023_1478_MOESM1_ESM.zip › original image for blots/original data files Figure 4E hMOF.tif]

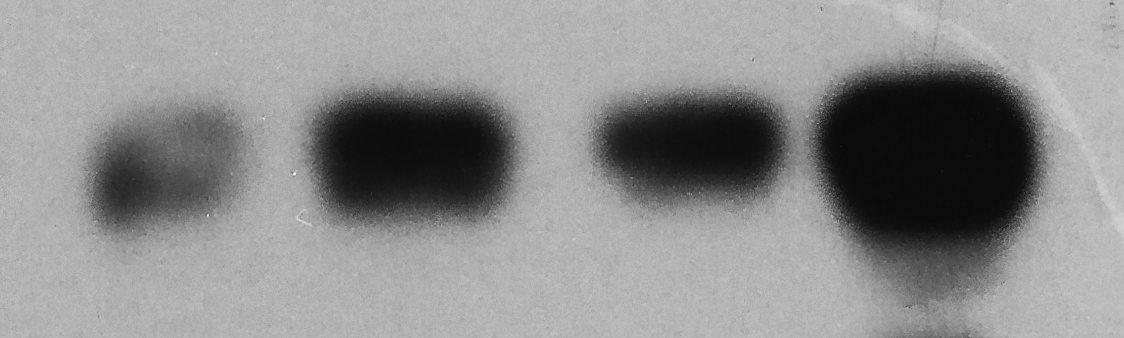

Supplement: Supplementary file 1 — original image for blots [file 41420_2023_1478_MOESM1_ESM.zip › original image for blots/original data files Figure 4E MDM2.tif]

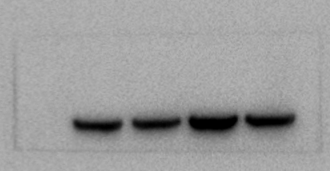

Supplement: Supplementary file 1 — original image for blots [file 41420_2023_1478_MOESM1_ESM.zip › original image for blots/original data files Figure 4E p53.tif]

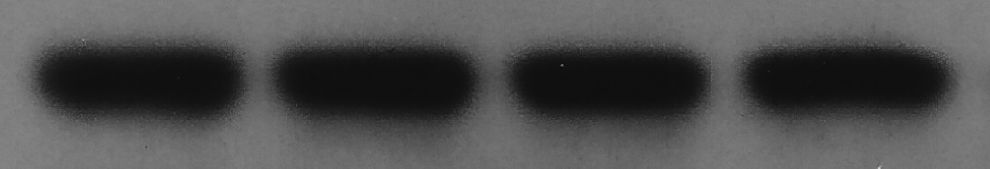

Supplement: Supplementary file 1 — original image for blots [file 41420_2023_1478_MOESM1_ESM.zip › original image for blots/original data files Figure 4E a┬-actin.tif]

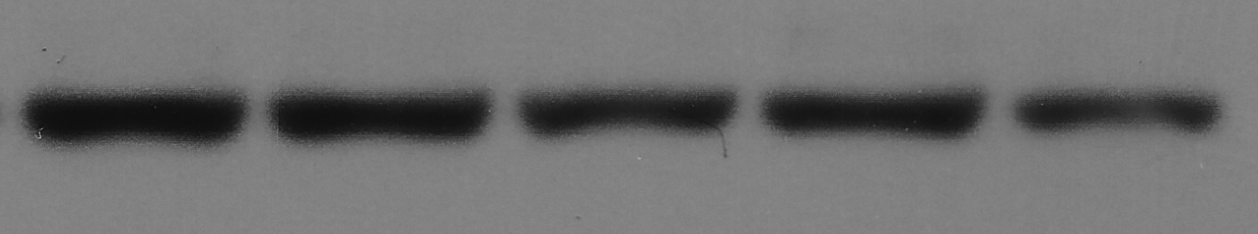

Supplement: Supplementary file 1 — original image for blots [file 41420_2023_1478_MOESM1_ESM.zip › original image for blots/original data files Figure 5A hMOF-hMOF.tif]

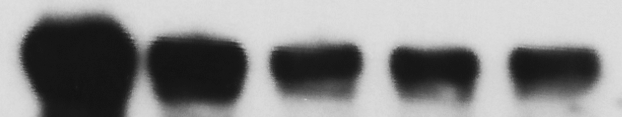

Supplement: Supplementary file 1 — original image for blots [file 41420_2023_1478_MOESM1_ESM.zip › original image for blots/original data files Figure 5A hMOF-MDM2.tif]

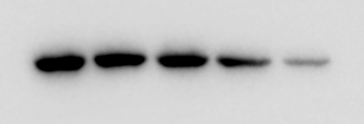

Supplement: Supplementary file 1 — original image for blots [file 41420_2023_1478_MOESM1_ESM.zip › original image for blots/original data files Figure 5A hMOF-p53.tif]

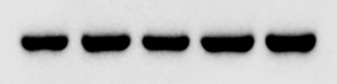

Supplement: Supplementary file 1 — original image for blots [file 41420_2023_1478_MOESM1_ESM.zip › original image for blots/original data files Figure 5A hMOF-a┬-actin.tif]

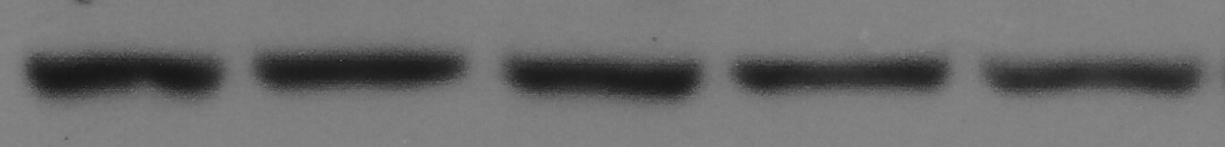

Supplement: Supplementary file 1 — original image for blots [file 41420_2023_1478_MOESM1_ESM.zip › original image for blots/original data files Figure 5A NC-hMOF.tif]

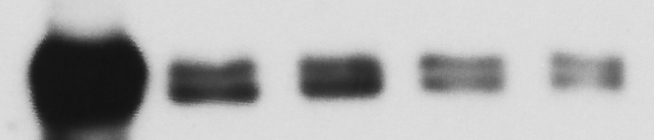

Supplement: Supplementary file 1 — original image for blots [file 41420_2023_1478_MOESM1_ESM.zip › original image for blots/original data files Figure 5A NC-MDM2.tif]

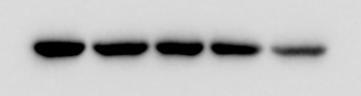

Supplement: Supplementary file 1 — original image for blots [file 41420_2023_1478_MOESM1_ESM.zip › original image for blots/original data files Figure 5A NC-p53.tif]

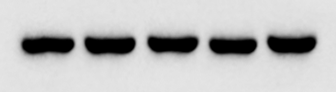

Supplement: Supplementary file 1 — original image for blots [file 41420_2023_1478_MOESM1_ESM.zip › original image for blots/original data files Figure 5A NC-a┬-actin.tif]

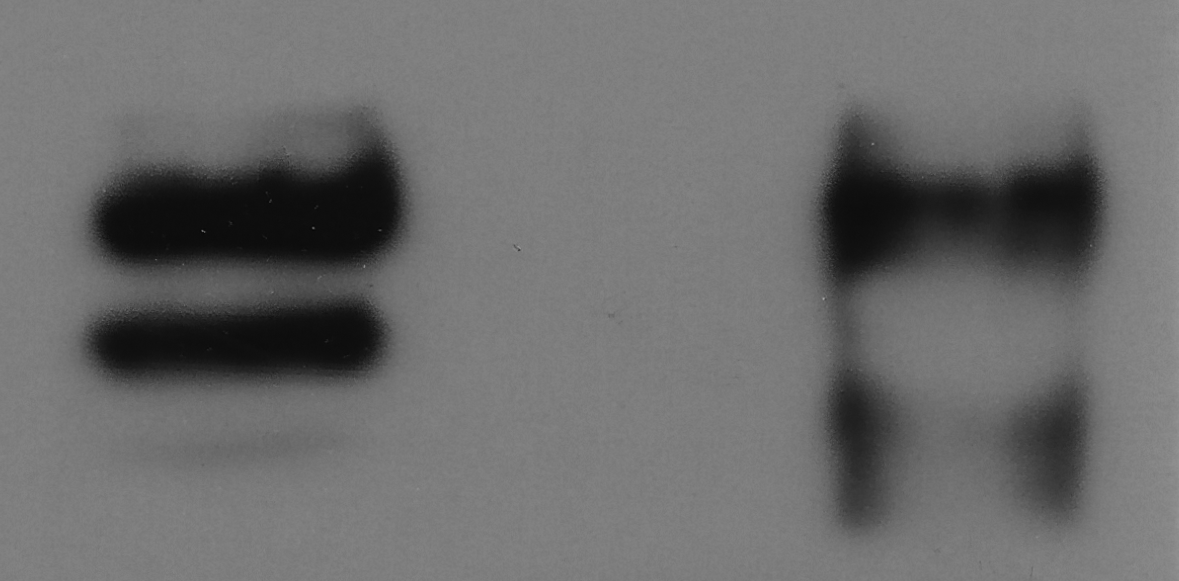

Supplement: Supplementary file 1 — original image for blots [file 41420_2023_1478_MOESM1_ESM.zip › original image for blots/original data files Figure 5B IB hMOF.tif]

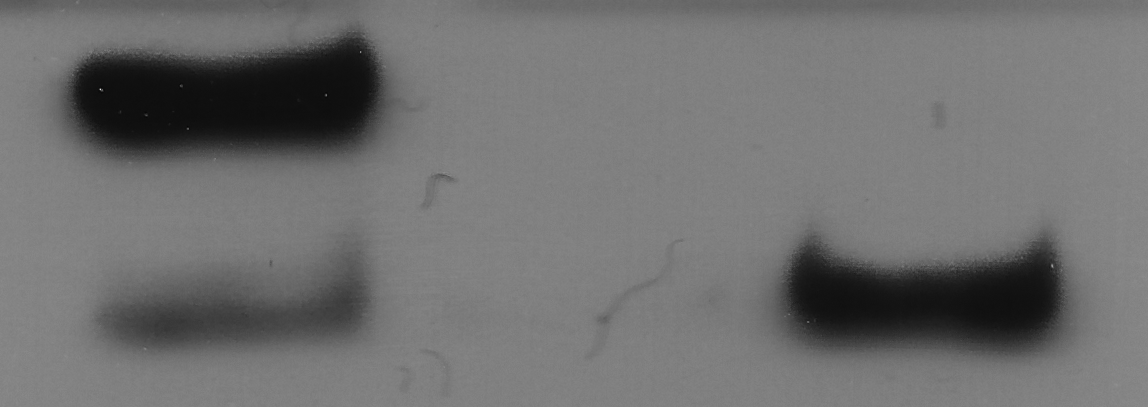

Supplement: Supplementary file 1 — original image for blots [file 41420_2023_1478_MOESM1_ESM.zip › original image for blots/original data files Figure 5B IB MDM2.tif]

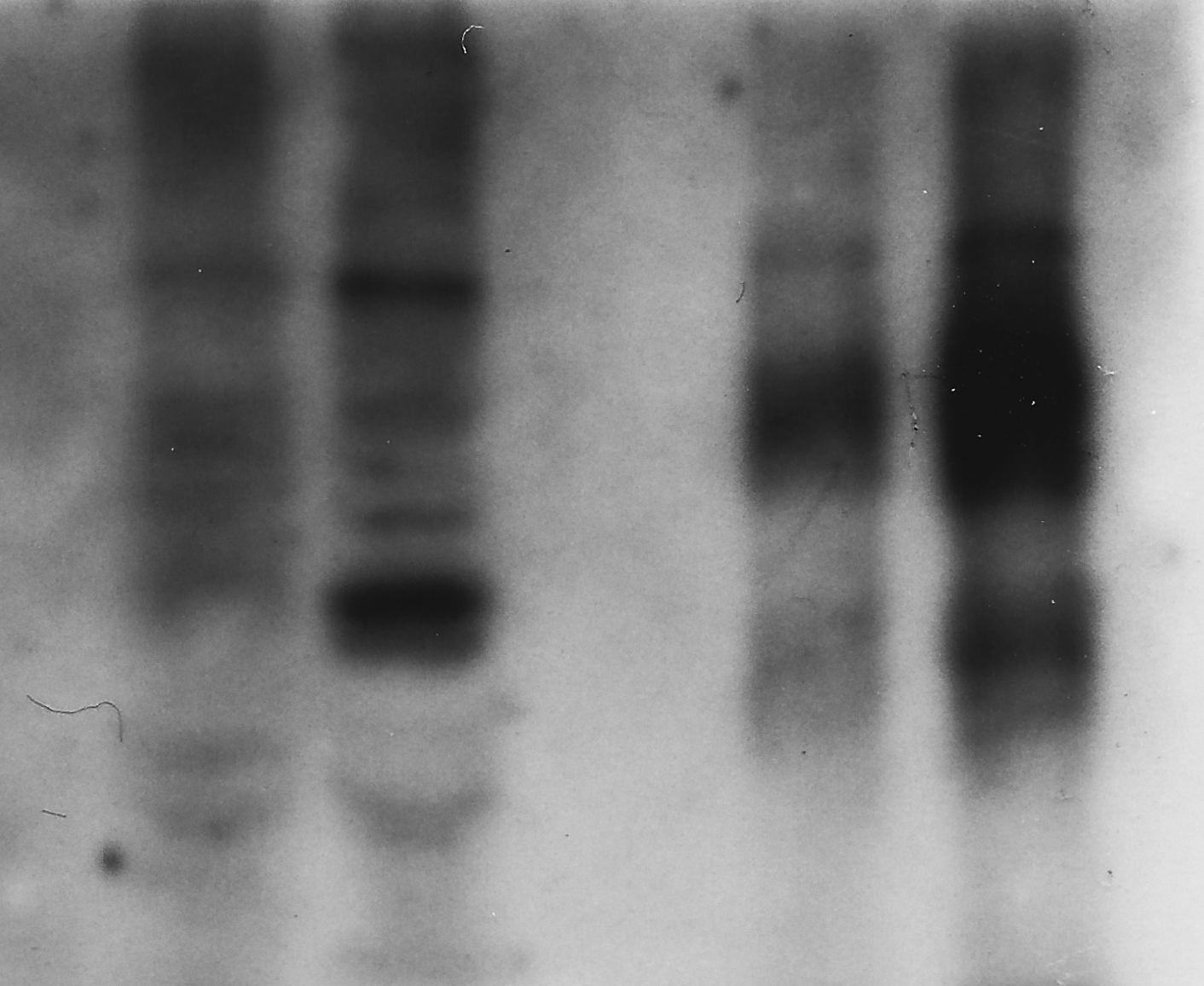

Supplement: Supplementary file 1 — original image for blots [file 41420_2023_1478_MOESM1_ESM.zip › original image for blots/original data files Figure 5D IB Acetylated-Lysine.tif]

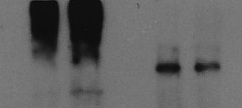

Supplement: Supplementary file 1 — original image for blots [file 41420_2023_1478_MOESM1_ESM.zip › original image for blots/original data files Figure 5D IB Ubiquitin.tif]

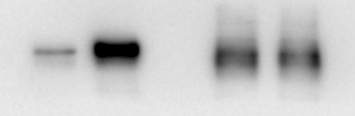

Supplement: Supplementary file 1 — original image for blots [file 41420_2023_1478_MOESM1_ESM.zip › original image for blots/original data files Figure 5D MDM2.tif]

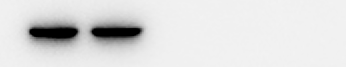

Supplement: Supplementary file 1 — original image for blots [file 41420_2023_1478_MOESM1_ESM.zip › original image for blots/original data files Figure 5D a┬-actin.tif]

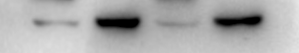

Supplement: Supplementary file 1 — original image for blots [file 41420_2023_1478_MOESM1_ESM.zip › original image for blots/original data files Figure 5E hMOF.tif]

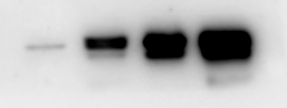

Supplement: Supplementary file 1 — original image for blots [file 41420_2023_1478_MOESM1_ESM.zip › original image for blots/original data files Figure 5E MDM2.tif]

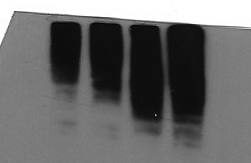

Supplement: Supplementary file 1 — original image for blots [file 41420_2023_1478_MOESM1_ESM.zip › original image for blots/original data files Figure 5E Ubiquitin.tif]

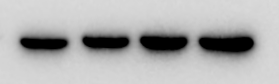

Supplement: Supplementary file 1 — original image for blots [file 41420_2023_1478_MOESM1_ESM.zip › original image for blots/original data files Figure 5E a┬-actin.tif]

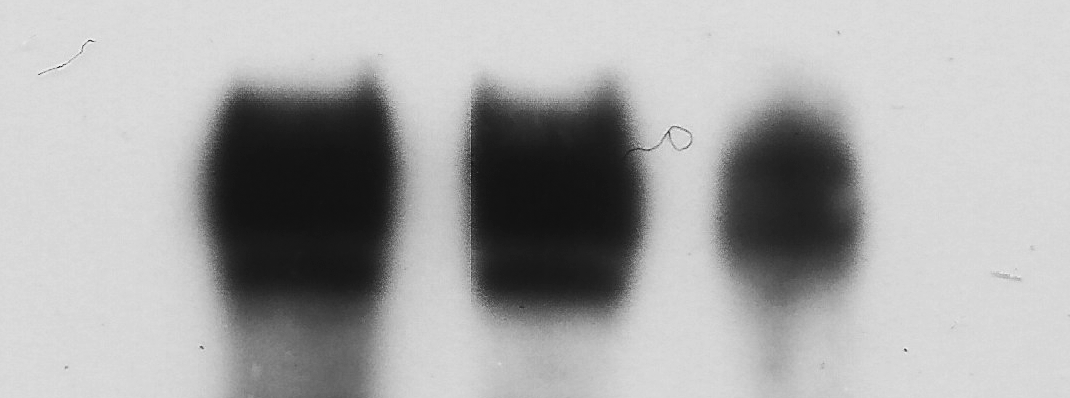

Supplement: Supplementary file 1 — original image for blots [file 41420_2023_1478_MOESM1_ESM.zip › original image for blots/original data files Figure 6A MDM2.tif]

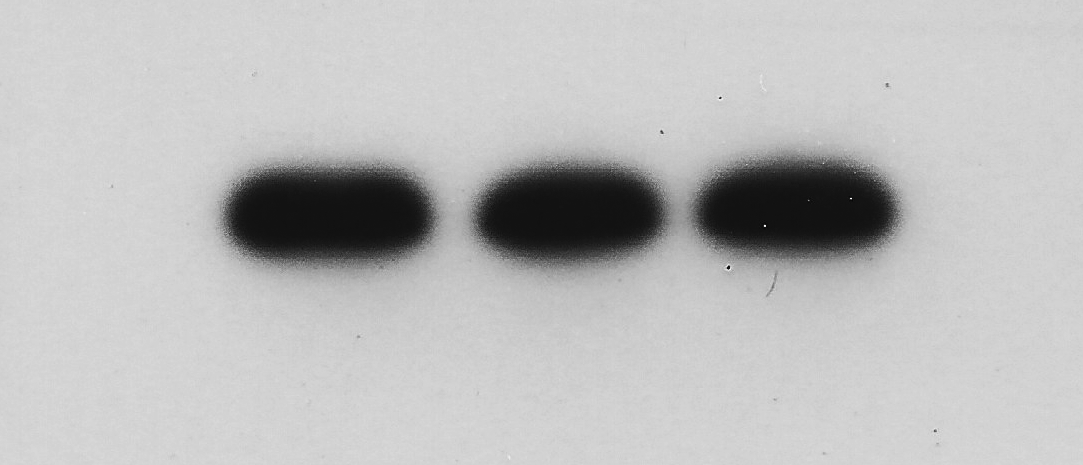

Supplement: Supplementary file 1 — original image for blots [file 41420_2023_1478_MOESM1_ESM.zip › original image for blots/original data files Figure 6A a┬-actin.tif]

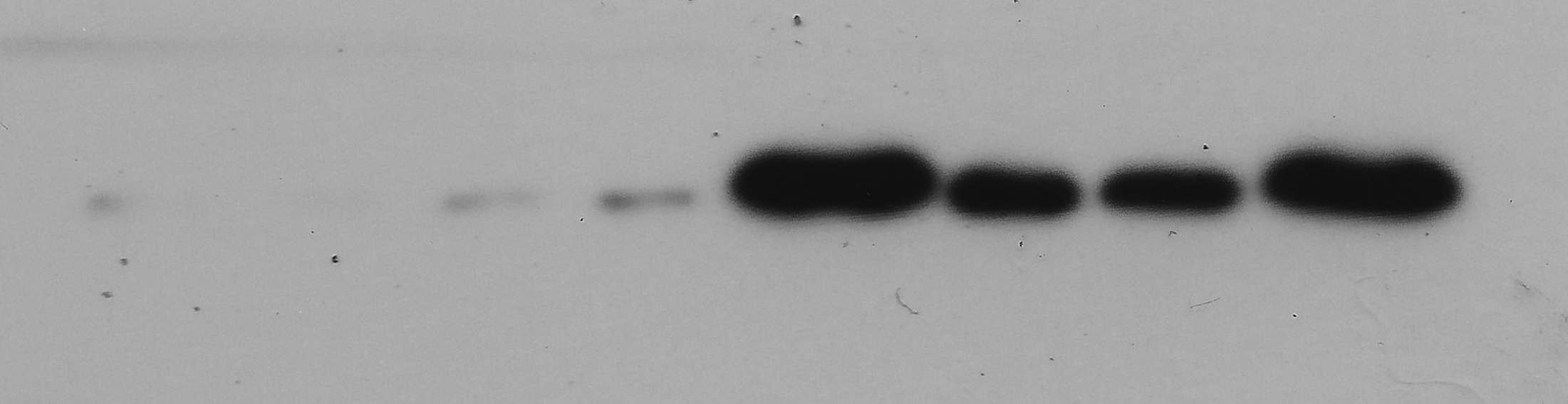

Supplement: Supplementary file 1 — original image for blots [file 41420_2023_1478_MOESM1_ESM.zip › original image for blots/original data files Figure 6D Bax.tif]

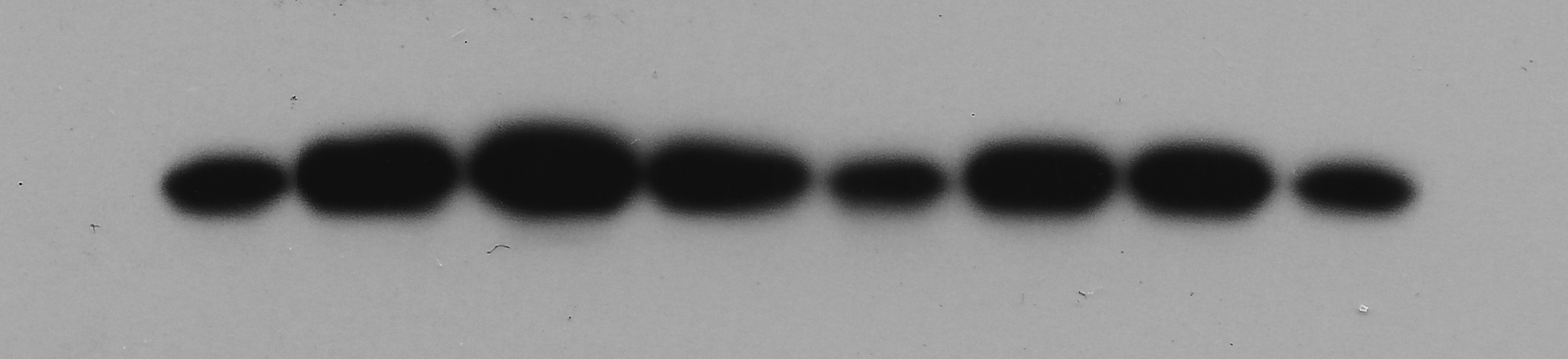

Supplement: Supplementary file 1 — original image for blots [file 41420_2023_1478_MOESM1_ESM.zip › original image for blots/original data files Figure 6D Bcl-2.tif]

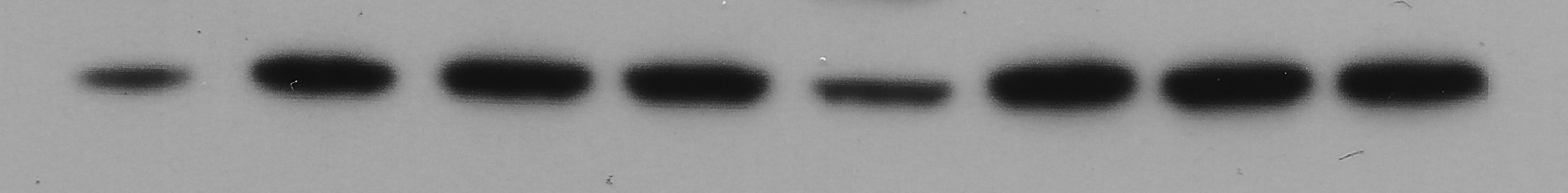

Supplement: Supplementary file 1 — original image for blots [file 41420_2023_1478_MOESM1_ESM.zip › original image for blots/original data files Figure 6D hMOF.tif]

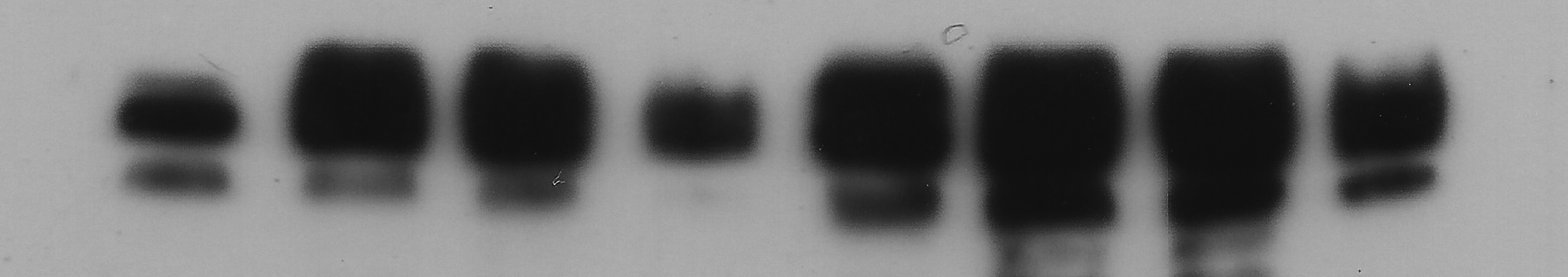

Supplement: Supplementary file 1 — original image for blots [file 41420_2023_1478_MOESM1_ESM.zip › original image for blots/original data files Figure 6D MDM2.tif]

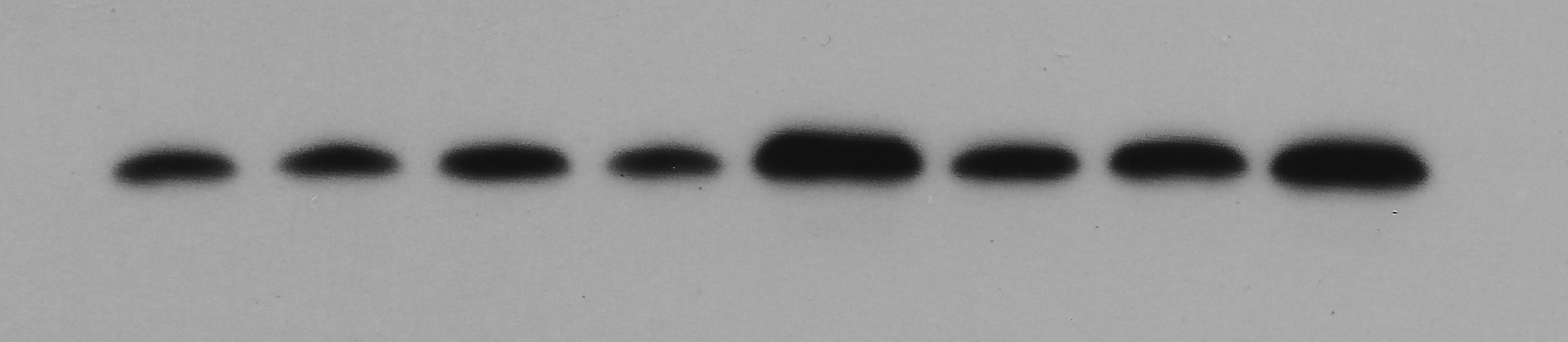

Supplement: Supplementary file 1 — original image for blots [file 41420_2023_1478_MOESM1_ESM.zip › original image for blots/original data files Figure 6D p53.tif]

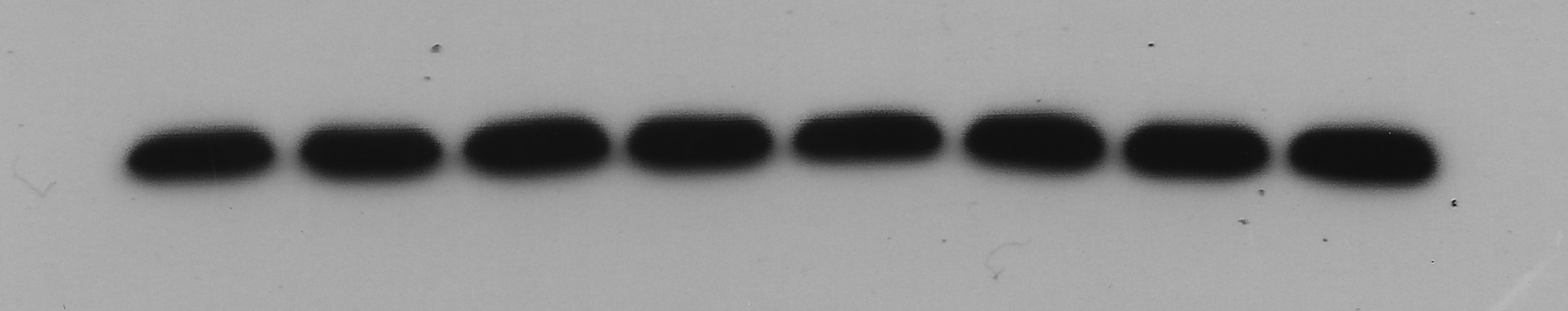

Supplement: Supplementary file 1 — original image for blots [file 41420_2023_1478_MOESM1_ESM.zip › original image for blots/original data files Figure 6D a┬-actin.tif]

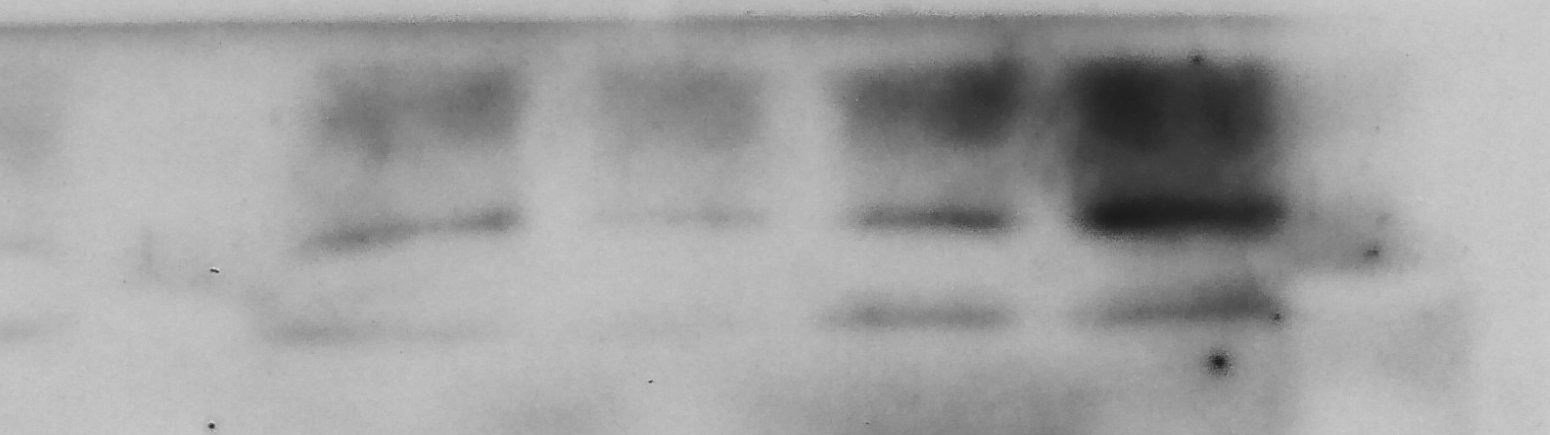

Supplement: Supplementary file 1 — original image for blots [file 41420_2023_1478_MOESM1_ESM.zip › original image for blots/original data files Figure 7D Bax.tif]

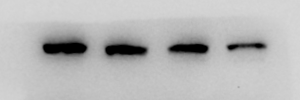

Supplement: Supplementary file 1 — original image for blots [file 41420_2023_1478_MOESM1_ESM.zip › original image for blots/original data files Figure 7D Bcl-2.tif]

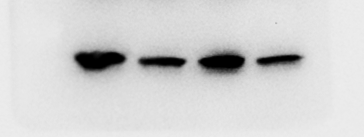

Supplement: Supplementary file 1 — original image for blots [file 41420_2023_1478_MOESM1_ESM.zip › original image for blots/original data files Figure 7D hMOF.tif]

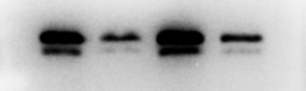

Supplement: Supplementary file 1 — original image for blots [file 41420_2023_1478_MOESM1_ESM.zip › original image for blots/original data files Figure 7D MDM2.tif]

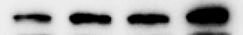

Supplement: Supplementary file 1 — original image for blots [file 41420_2023_1478_MOESM1_ESM.zip › original image for blots/original data files Figure 7D p53.tif]

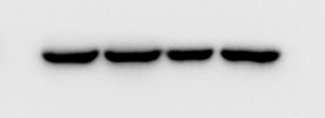

Supplement: Supplementary file 1 — original image for blots [file 41420_2023_1478_MOESM1_ESM.zip › original image for blots/original data files Figure 7D a┬-actin.tif]
