# Supplementary material for: hMOF induces cisplatin resistance of ovarian cancer by regulating the stability and expression of MDM2
Source: Cell Death Discov. 2023 Jun 8;9:179. doi: 10.1038/s41420-023-01478-y (PMC10250293; doi:10.1038/s41420-023-01478-y)
Supplement: Supplementary file 2 — supplementary table.1 [file 41420_2023_1478_MOESM2_ESM.docx]

Supplementary Table. 1

| Premiers | Sequence | |
| --- | --- | --- |
|  | Forward | Reverse |
| β-actin | CGGCACCACCATGTACCCTG | ACACGGAGTACTTGCGCTCA |
| hMOF | CAGCCAGATGACCAGTATCACC | GGCCCTTCCAGTACTTGACCA |
| MDM2 | TCAAGTTACTGTGTATCAGGC | GTGCATTTCCAATAGTCAGC |
| p53 | CCTCCTCAGCATCTTATCCGAGT | TGGTACAGTCAGAGCCAACCTCA |
| Bcl-2 | CGACGACTTCTCCCGCCGCTA | GACATCTCCCGGTTGACGCTCT |
| Bak | GCCACCAGCCTGTTTGAG | CTGCCACCCAGCCACCC |
| Bid | TGGTGTTTGGCTTCCTCCAA | GAATCTGCCTCTATTCTTCCC |
| Noxa | TGGAAGTCGAGTGTGCTACTCAAC | CAGAAGAGTTTGGATATCAGATTCAGA |
| Fas | ATCCTGAAACAGTGGCAAT | ATGCCAATTACGAAGCAG |
| Nanog | CCCCAGCCTTTACTCTTCCTA | CCAGGTTGAATTGTTCCAGGTC |
| OCT4 | CAAAGCAGAAACCCTCGTGC | TCTCACTCGGTTCTCGATACTG |
| SOX2 | TGGACAGTTACGCGCACAT | CGAGTAGGACATGCTGTAGGT |
